# Supplementary figures and images for: The denitrosylase SCoR2 controls cardioprotective metabolic reprogramming
Source: eLife. 2025 Nov 17;14:RP106601. doi: 10.7554/eLife.106601 (PMC12622967; doi:10.7554/eLife.106601)

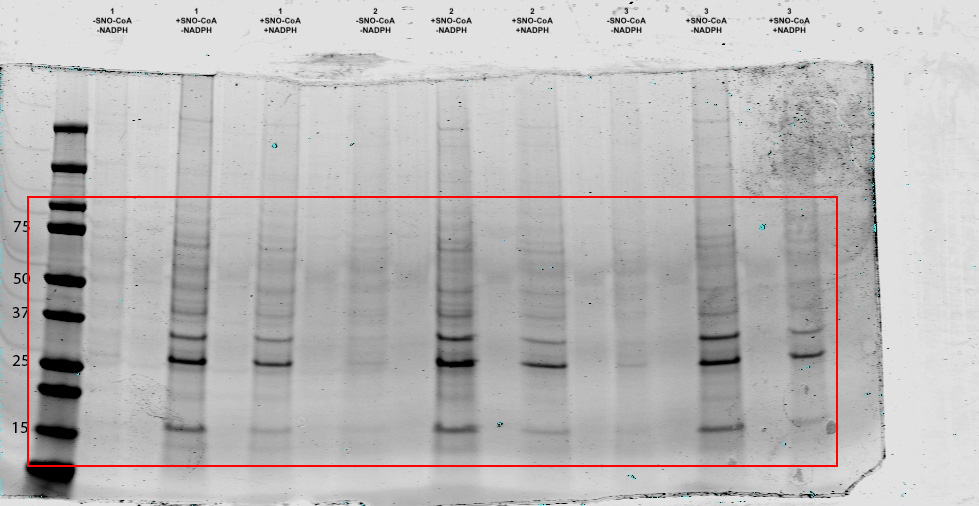

Supplement: Figure 2—source data 1. [file elife-106601-fig2-data1.zip › Figure 2-source data 1 (fig2D)/6_16_23 last 3 hearts NADPH SNO CoA SNO asc 2.jpg]

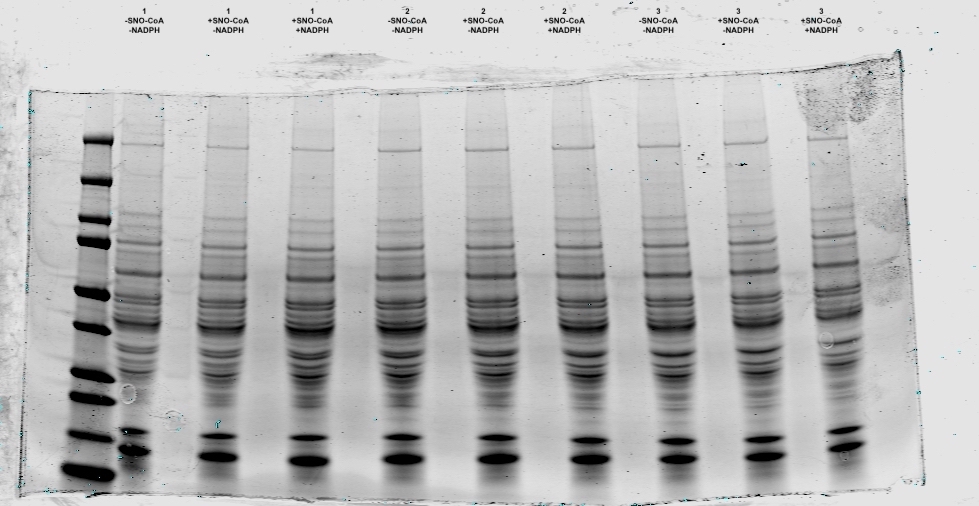

Supplement: Figure 2—source data 1. [file elife-106601-fig2-data1.zip › Figure 2-source data 1 (fig2D)/6_16_23last 3 hearts NADPH SNO CoA input (not shown).jpg]

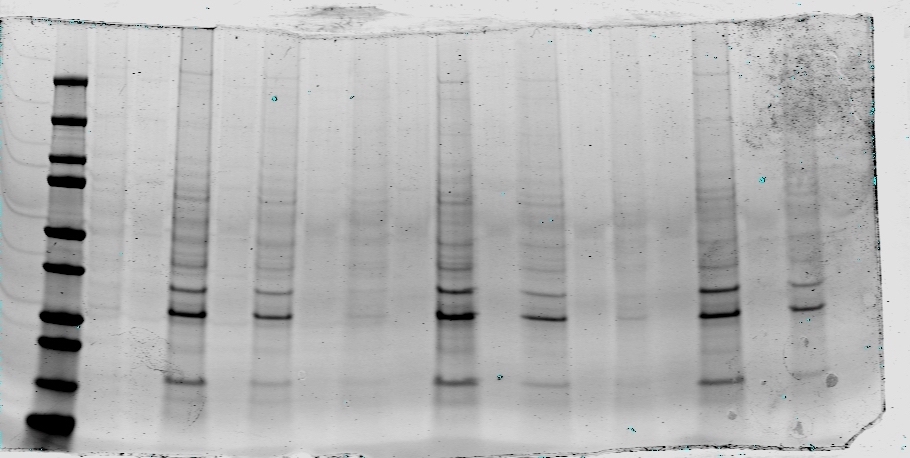

Supplement: Figure 2—source data 2. [file elife-106601-fig2-data2.zip › Figure 2-source data 2 (fig2D)/6_16_23 last 3 hearts NADPH SNO CoA SNO asc 2.jpg]

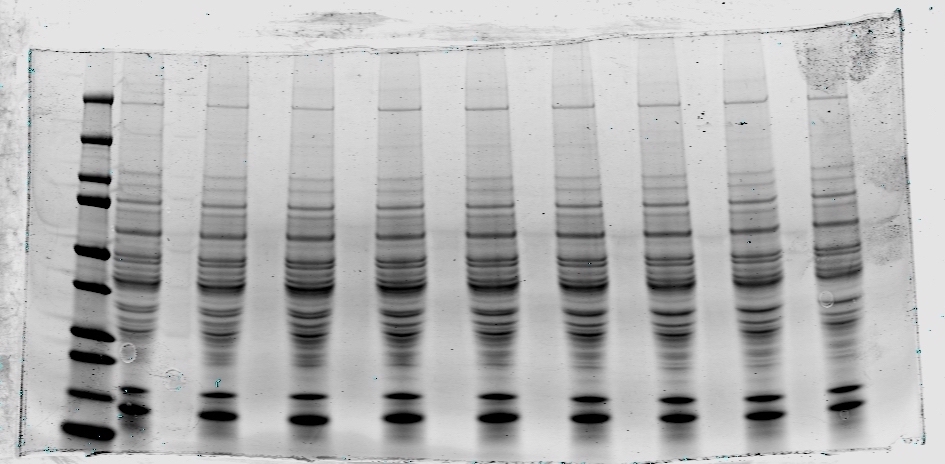

Supplement: Figure 2—source data 2. [file elife-106601-fig2-data2.zip › Figure 2-source data 2 (fig2D)/6_16_23last 3 hearts NADPH SNO CoA input (not shown).jpg]

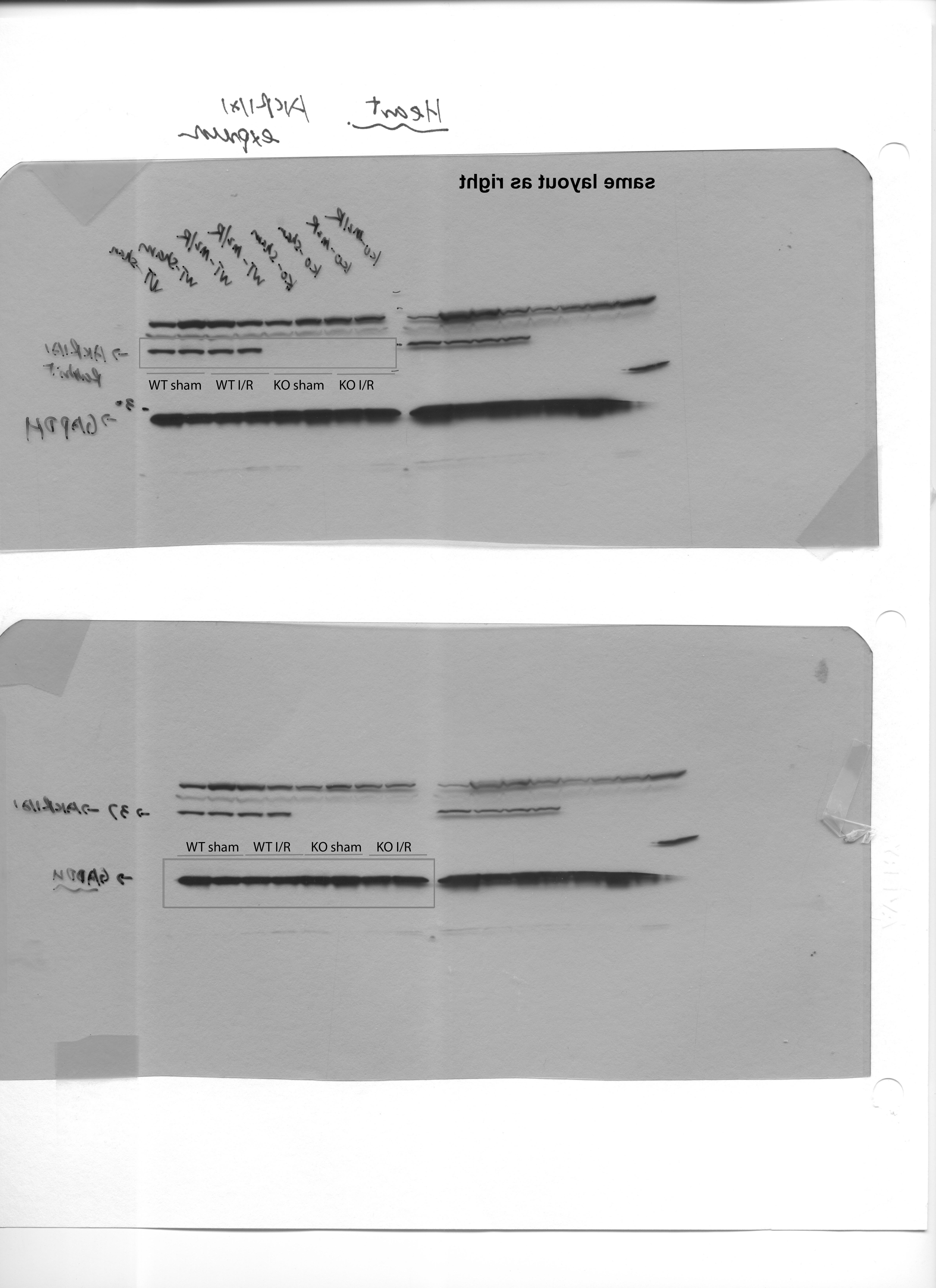

Supplement: Figure 2—figure supplement 1—source data 1. [file elife-106601-fig2-figsupp1-data1.zip › Figure 2-figure supplement 1-source data 1 (figS2B)/SCoR2 GAPDH S2B.jpg]

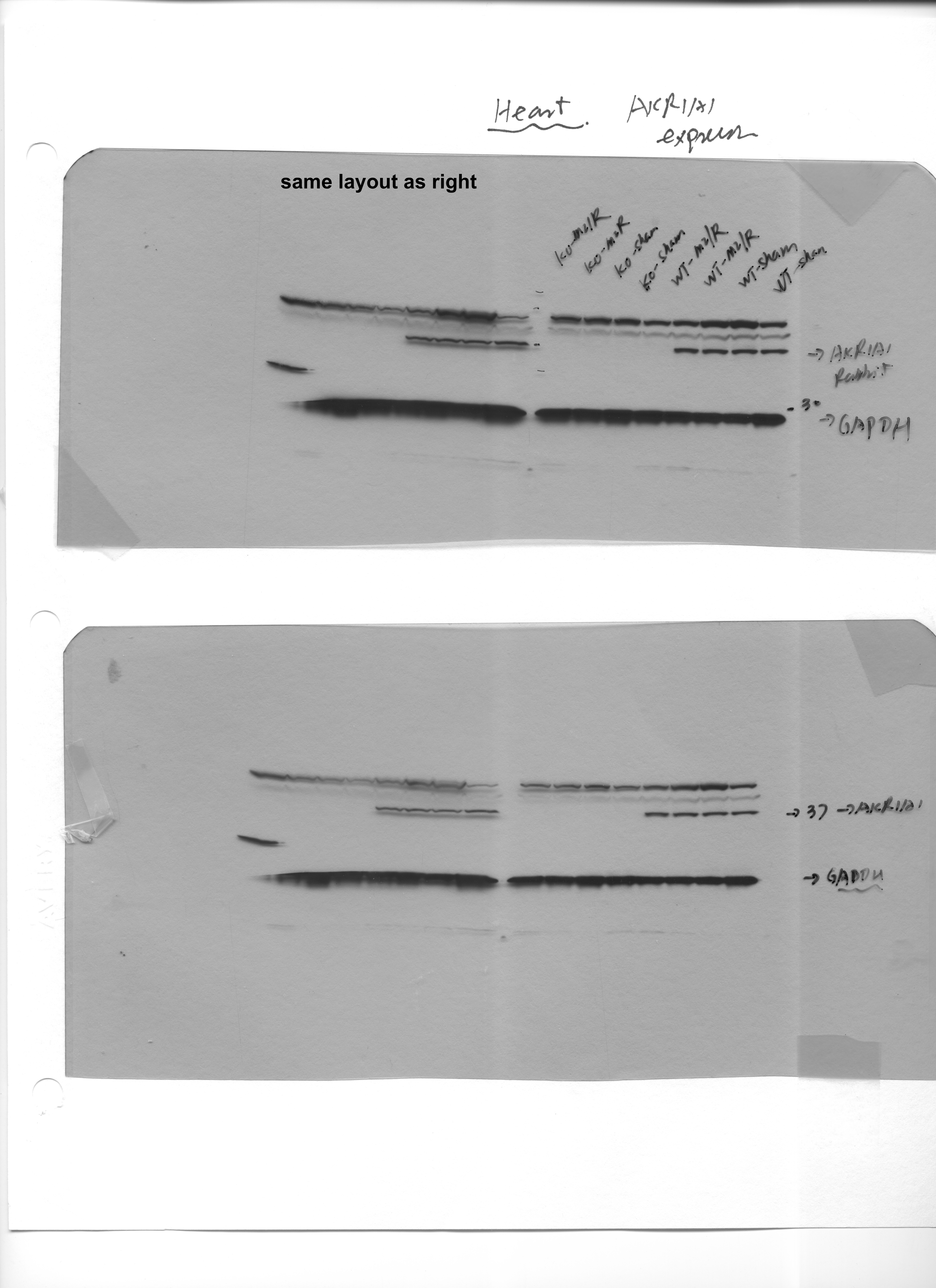

Supplement: Figure 2—figure supplement 1—source data 2. [file elife-106601-fig2-figsupp1-data2.zip › Figure 2-figure supplement 1-source data 2 (figS2B)/SCoR2 GAPDH S2B.jpg]

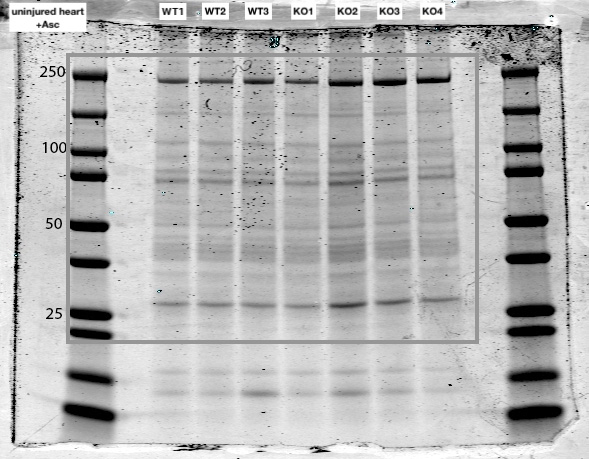

Supplement: Figure 2—figure supplement 1—source data 3. [file elife-106601-fig2-figsupp1-data3.zip › Figure 2-figure supplement 1-source data 3 (figS2D)/S2D.jpg]

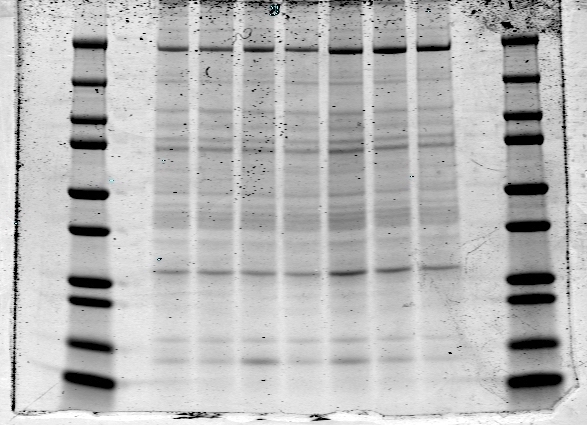

Supplement: Figure 2—figure supplement 1—source data 4. [file elife-106601-fig2-figsupp1-data4.zip › Figure 2-figure supplement 1-source data 4 (figS2D)/S2D.jpg]

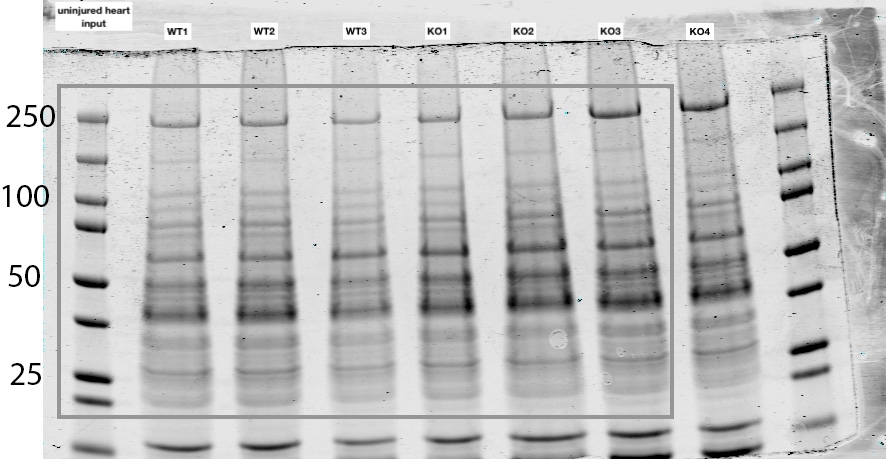

Supplement: Figure 2—figure supplement 1—source data 5. [file elife-106601-fig2-figsupp1-data5.zip › Figure 2-figure supplement 1-source data 5 (figS2E)/S2E.jpg]

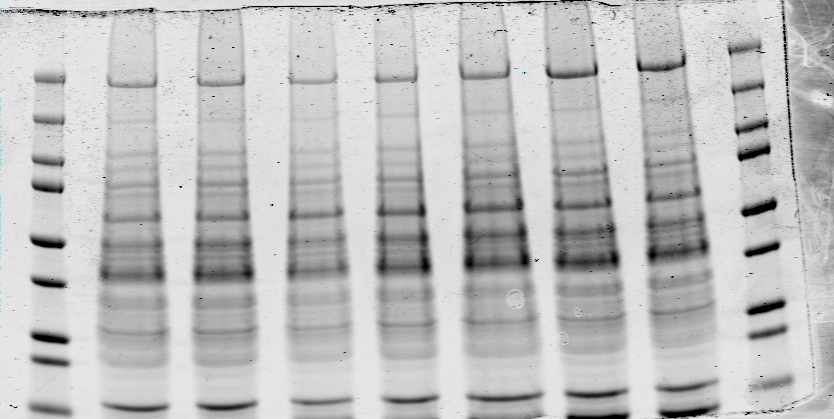

Supplement: Figure 2—figure supplement 1—source data 6. [file elife-106601-fig2-figsupp1-data6.zip › Figure 2-figure supplement 1-source data 6 (figS2E)/6_6_24 heart snorac input second run Fig S2E.jpg]

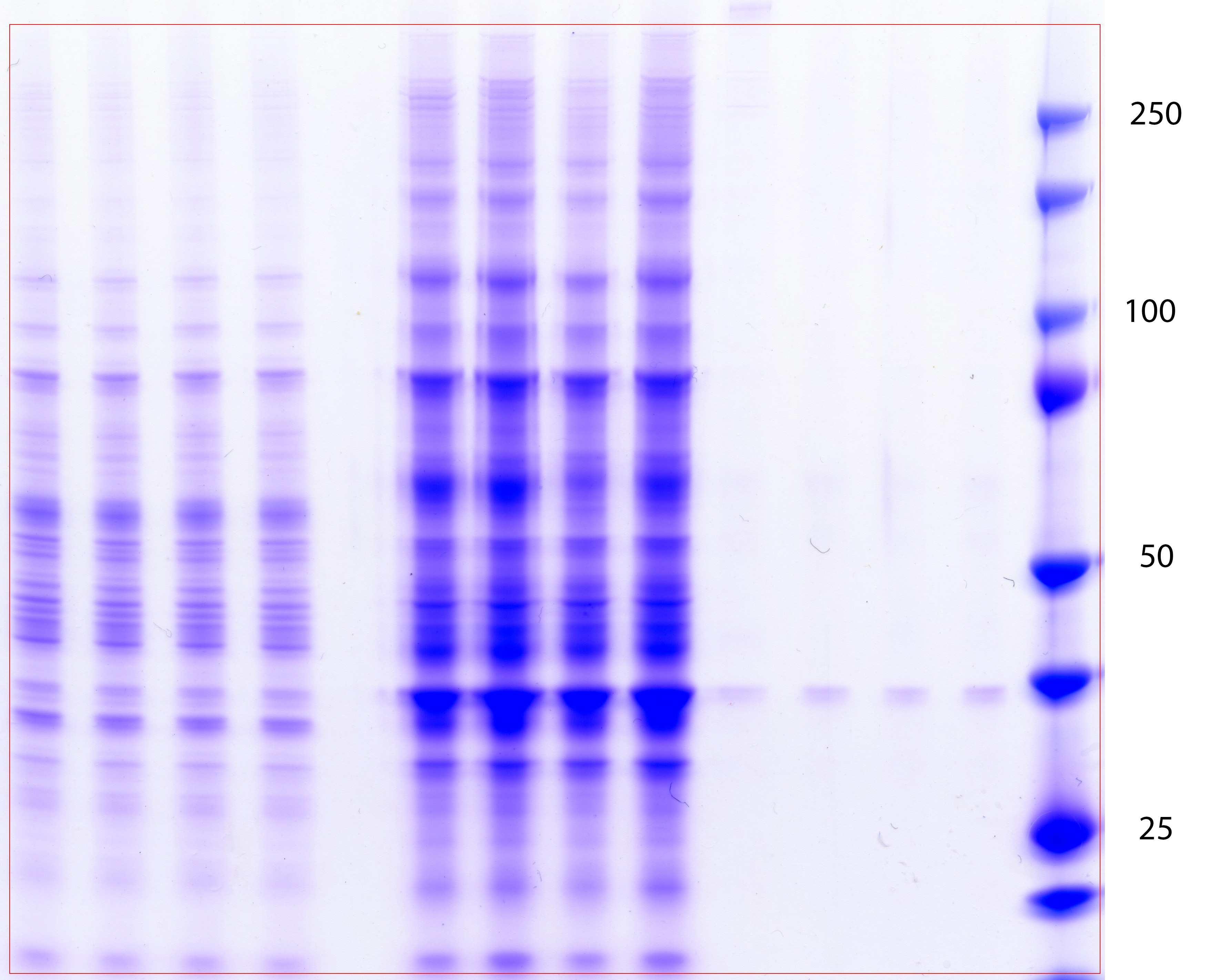

Supplement: Figure 3—source data 1. [file elife-106601-fig3-data1.zip › Source data (labeled) for Fig 3/Figure 3-source data 1 (fig3A)/MI-SNO from Hualin.jpg]

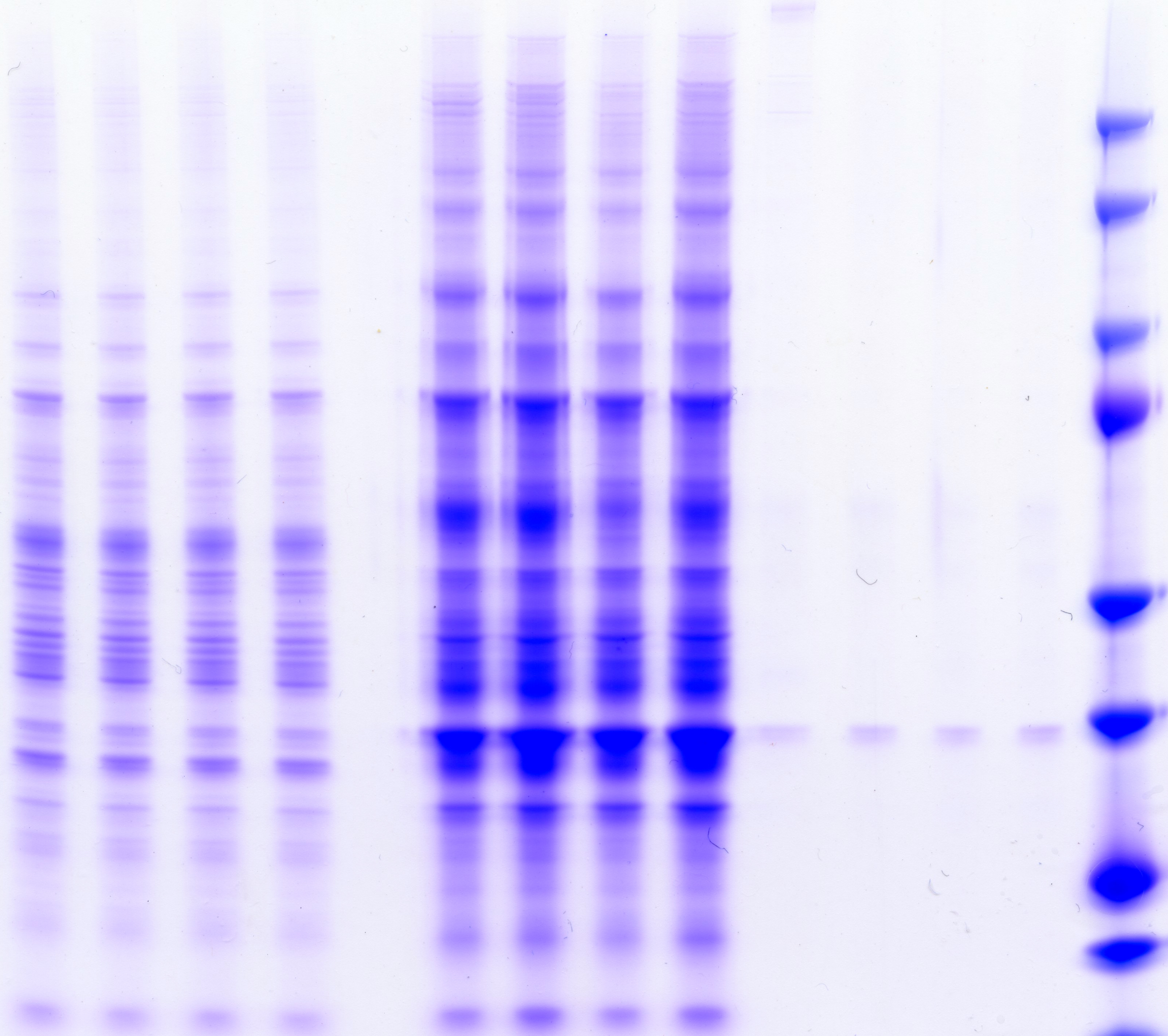

Supplement: Figure 3—source data 2. [file elife-106601-fig3-data2.zip › Source data (raw) for Fig 3/Figure 3-source data 2 (fig3A)/MI-SNO from Hualin.jpg]

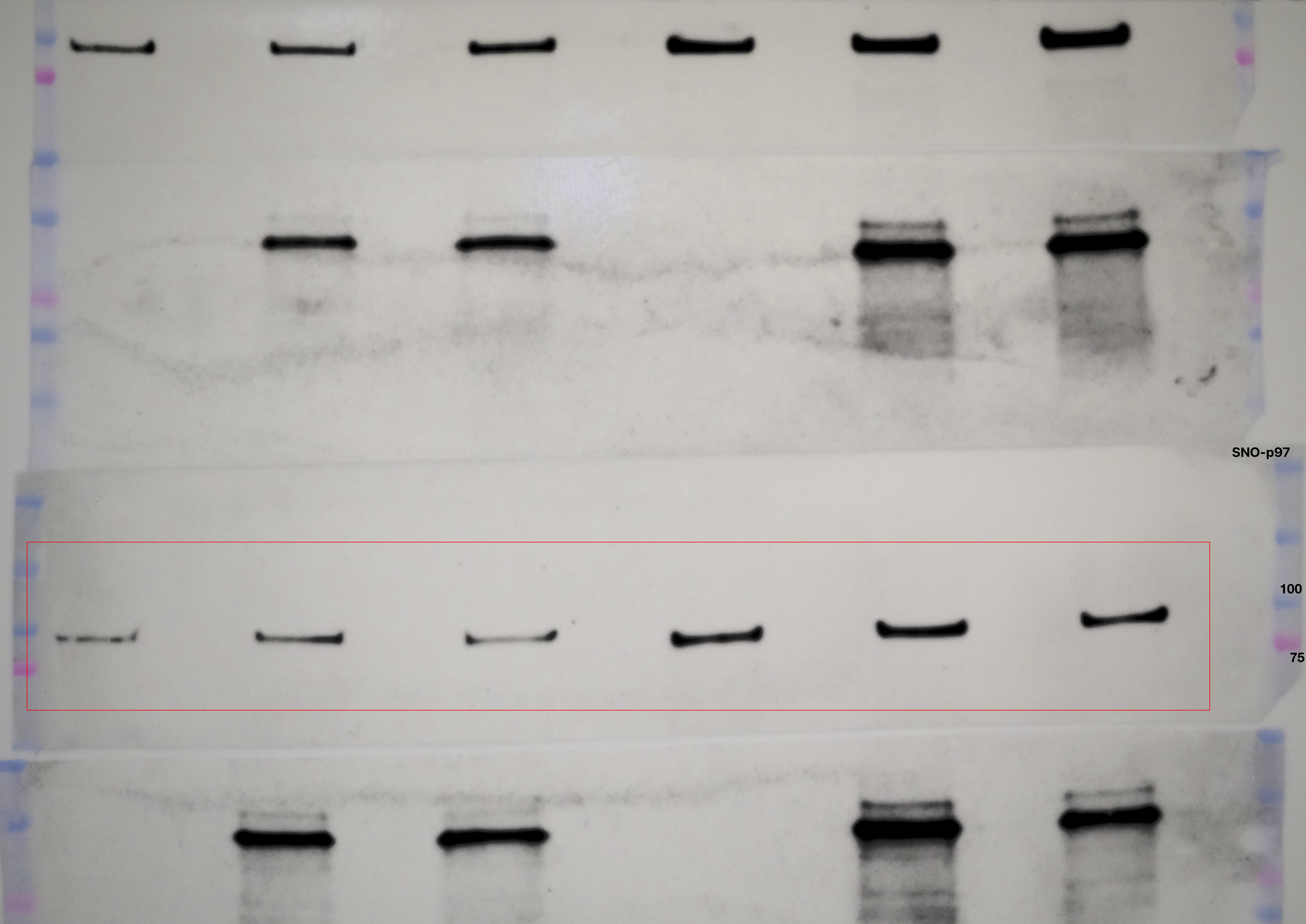

Supplement: Figure 4—source data 1. [file elife-106601-fig4-data1.zip › Figure 4-source data 1 (fig4G)/SNO-p97 Fig 4G.png]

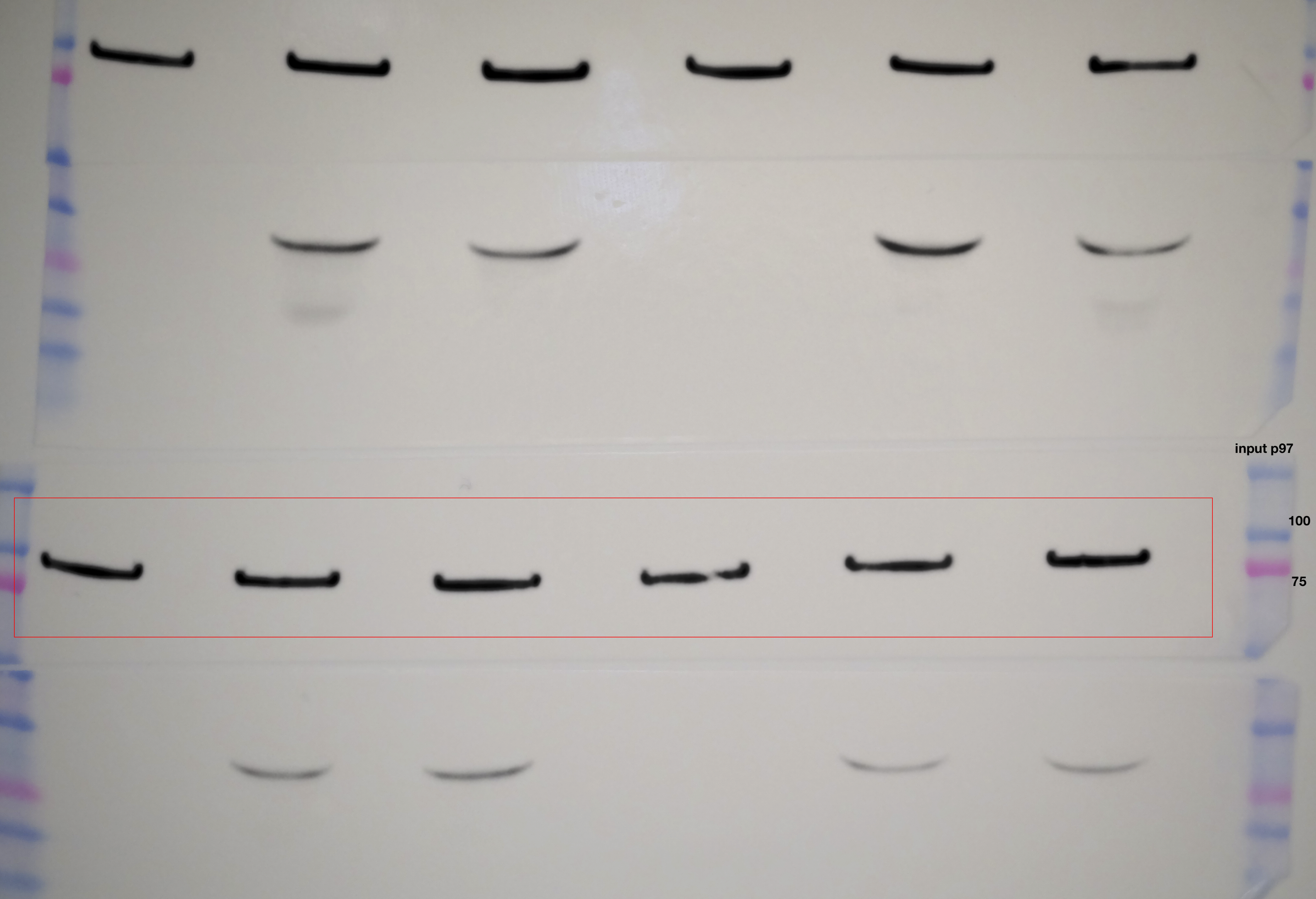

Supplement: Figure 4—source data 1. [file elife-106601-fig4-data1.zip › Figure 4-source data 1 (fig4G)/input p97 Fig 4G.png]

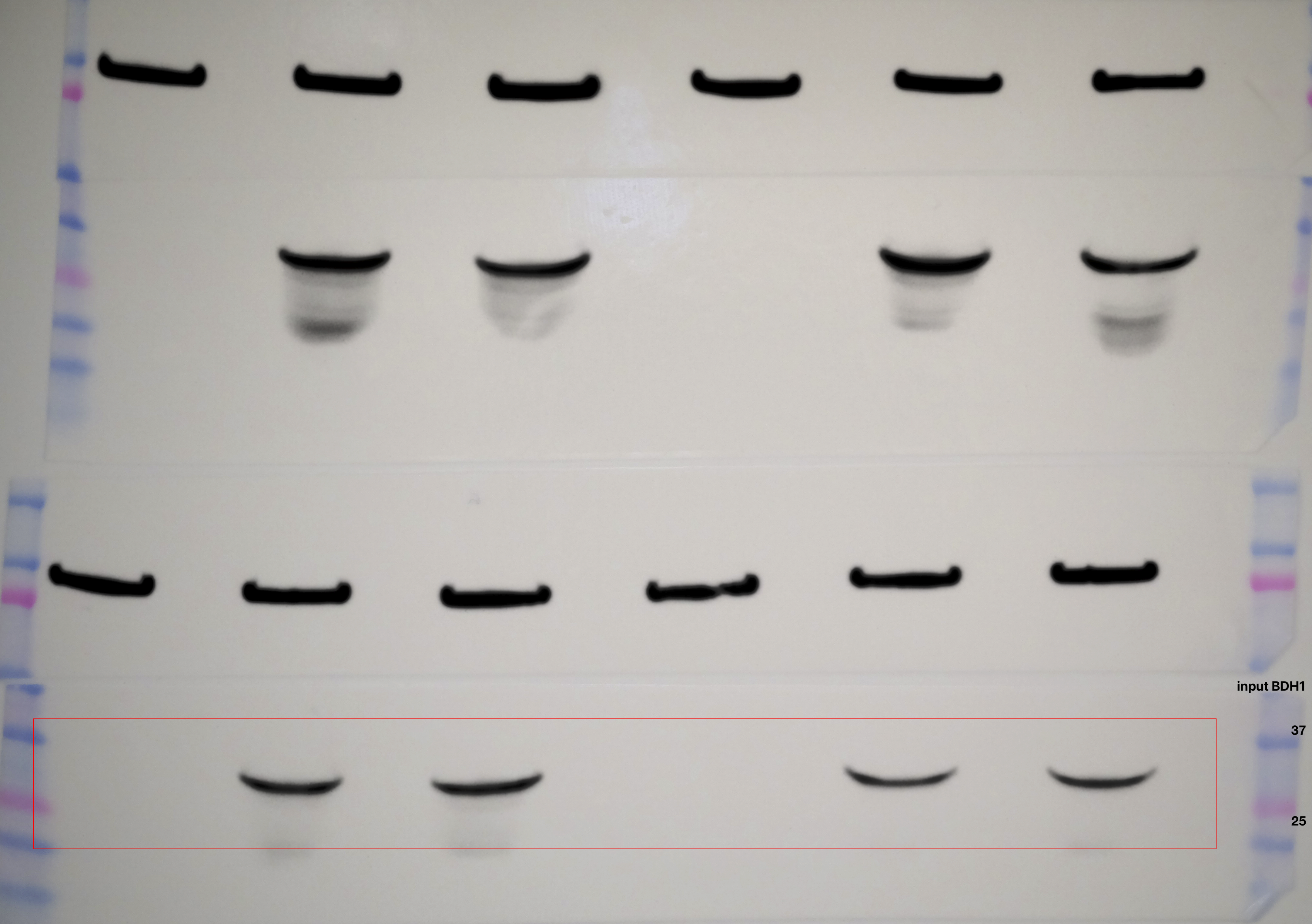

Supplement: Figure 4—source data 1. [file elife-106601-fig4-data1.zip › Figure 4-source data 1 (fig4G)/input BDH1 Fig 4G.png]

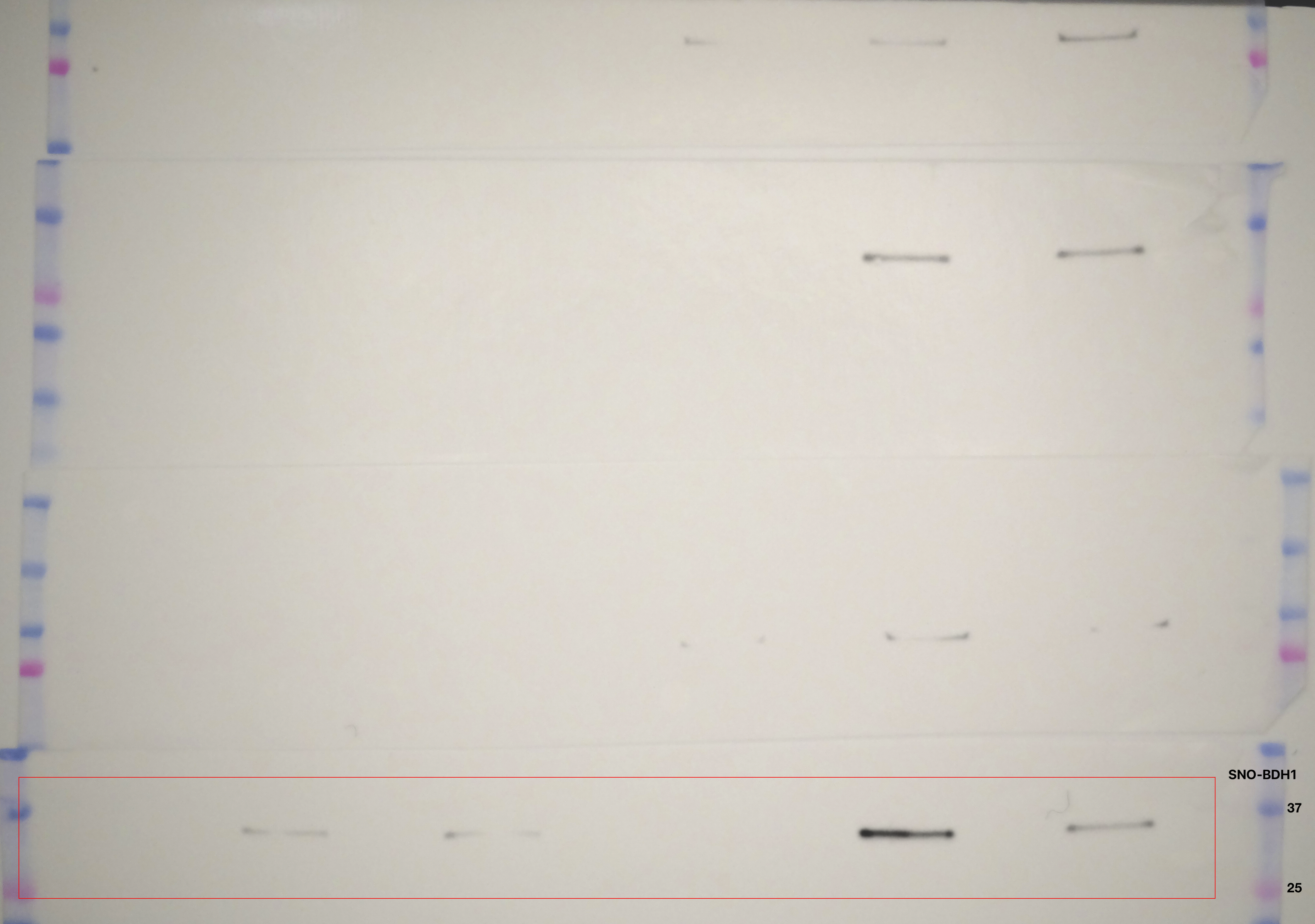

Supplement: Figure 4—source data 1. [file elife-106601-fig4-data1.zip › Figure 4-source data 1 (fig4G)/SNO-BDH1 Fig 4G.png]

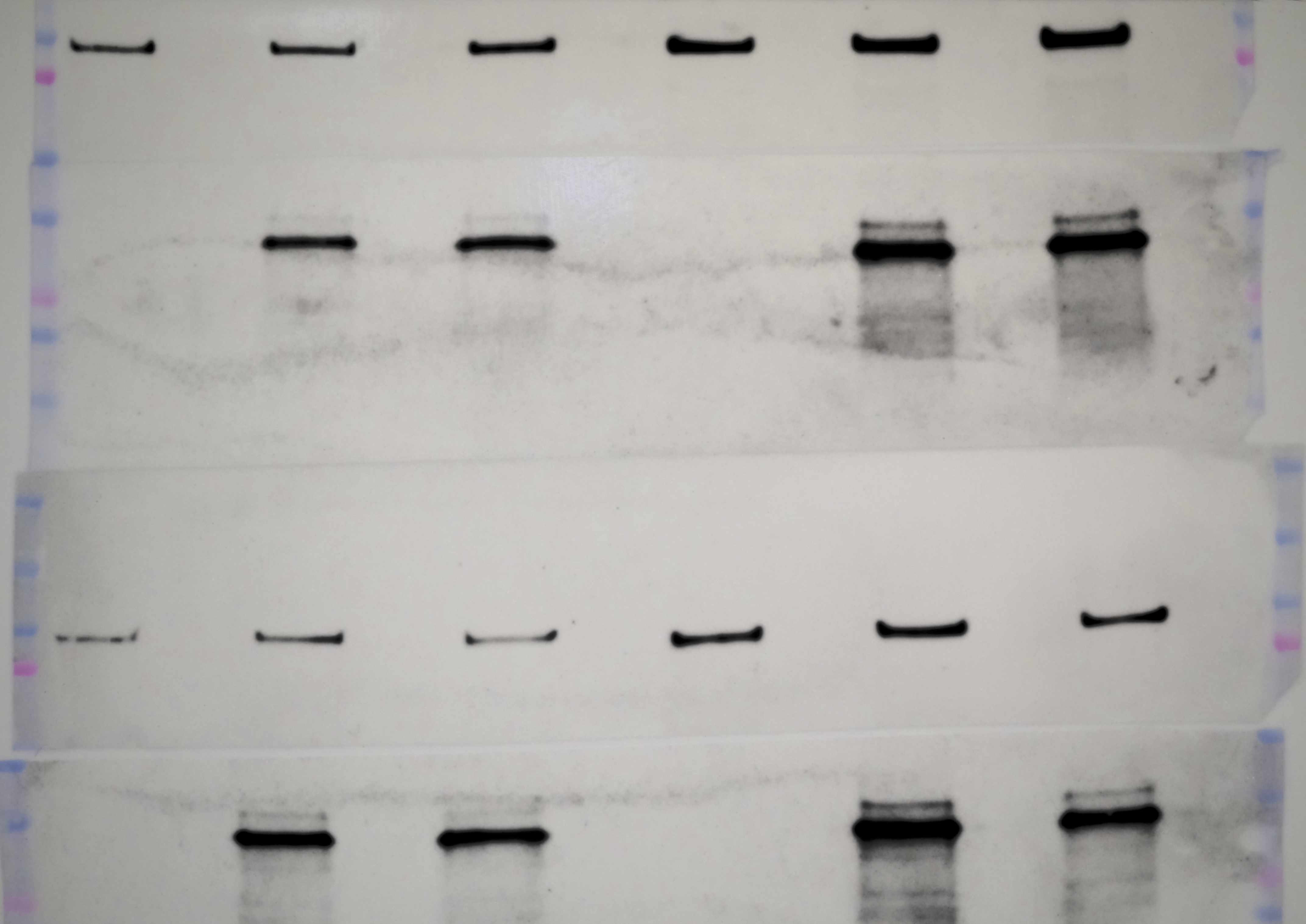

Supplement: Figure 4—source data 2. [file elife-106601-fig4-data2.zip › Figure 4-source data 2 (fig4G)/SNO-p97 Fig 4G.png]

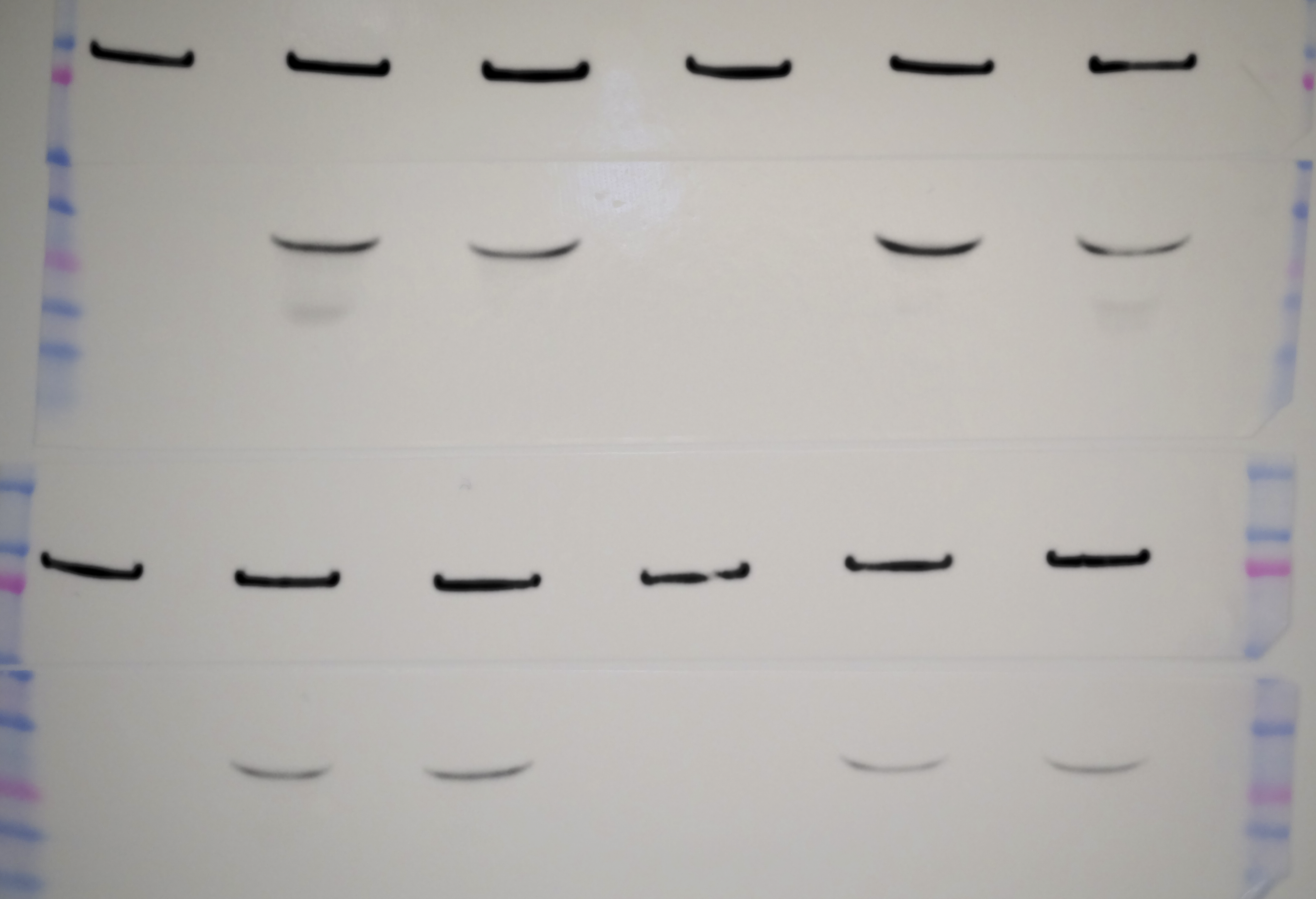

Supplement: Figure 4—source data 2. [file elife-106601-fig4-data2.zip › Figure 4-source data 2 (fig4G)/input p97 Fig 4G.png]

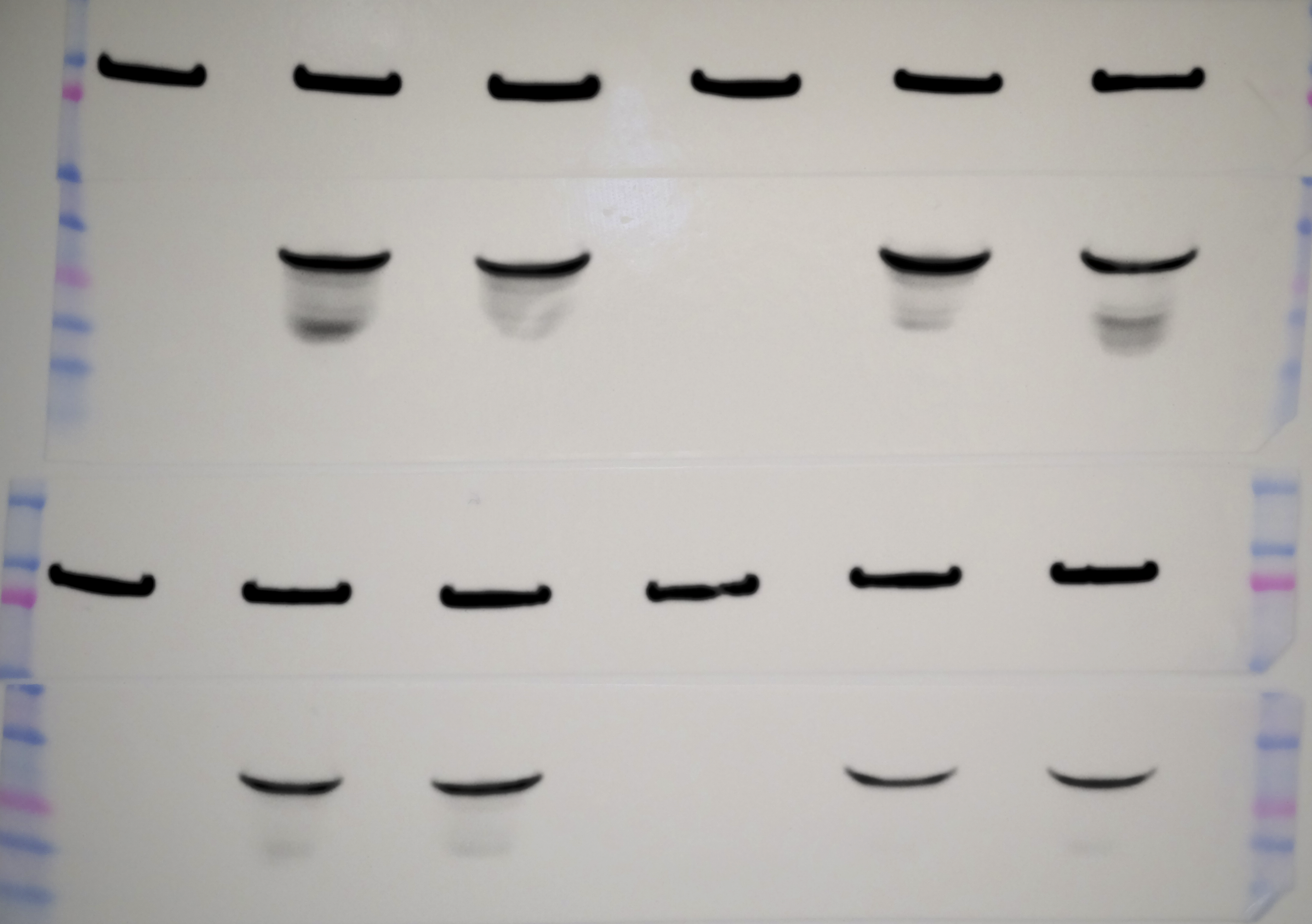

Supplement: Figure 4—source data 2. [file elife-106601-fig4-data2.zip › Figure 4-source data 2 (fig4G)/input bdh1 fig 4G.png]

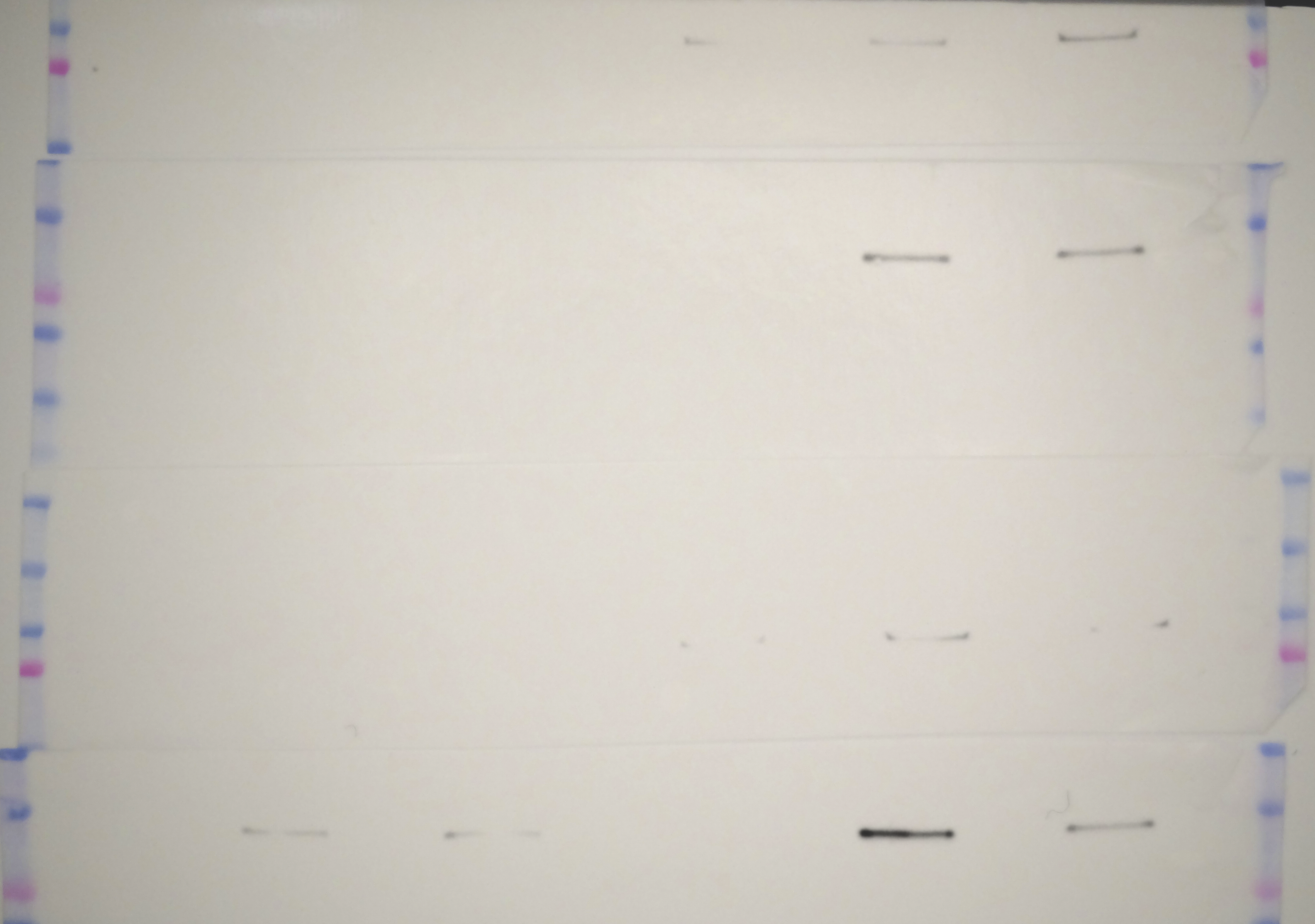

Supplement: Figure 4—source data 2. [file elife-106601-fig4-data2.zip › Figure 4-source data 2 (fig4G)/SNO-BDH1 Fig 4G.png]

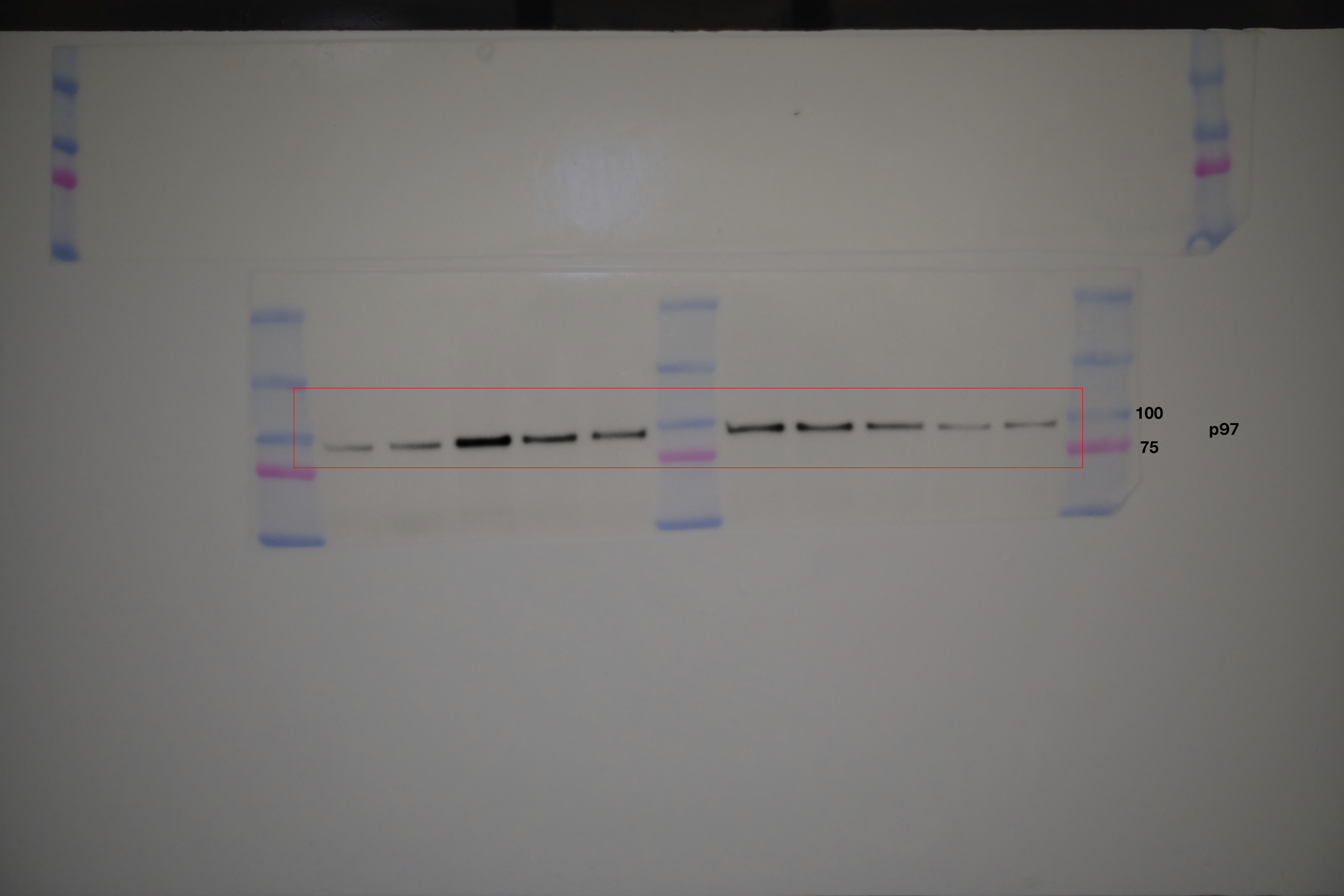

Supplement: Figure 4—source data 3. [file elife-106601-fig4-data3.zip › Figure 4-source data 3 (fig4O)/SNO-p97 4O.png]

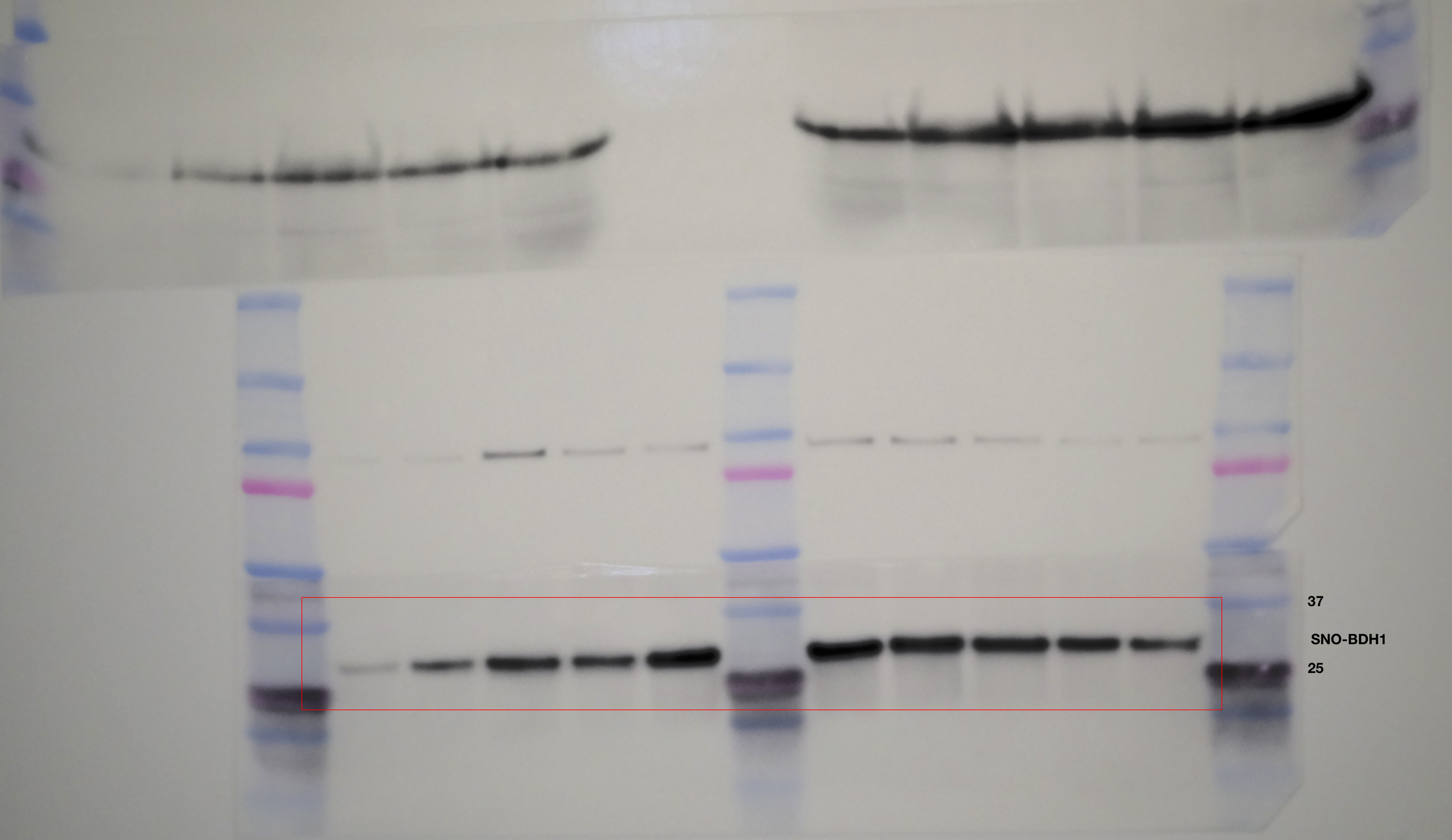

Supplement: Figure 4—source data 3. [file elife-106601-fig4-data3.zip › Figure 4-source data 3 (fig4O)/SNO-BDH1 4O.png]

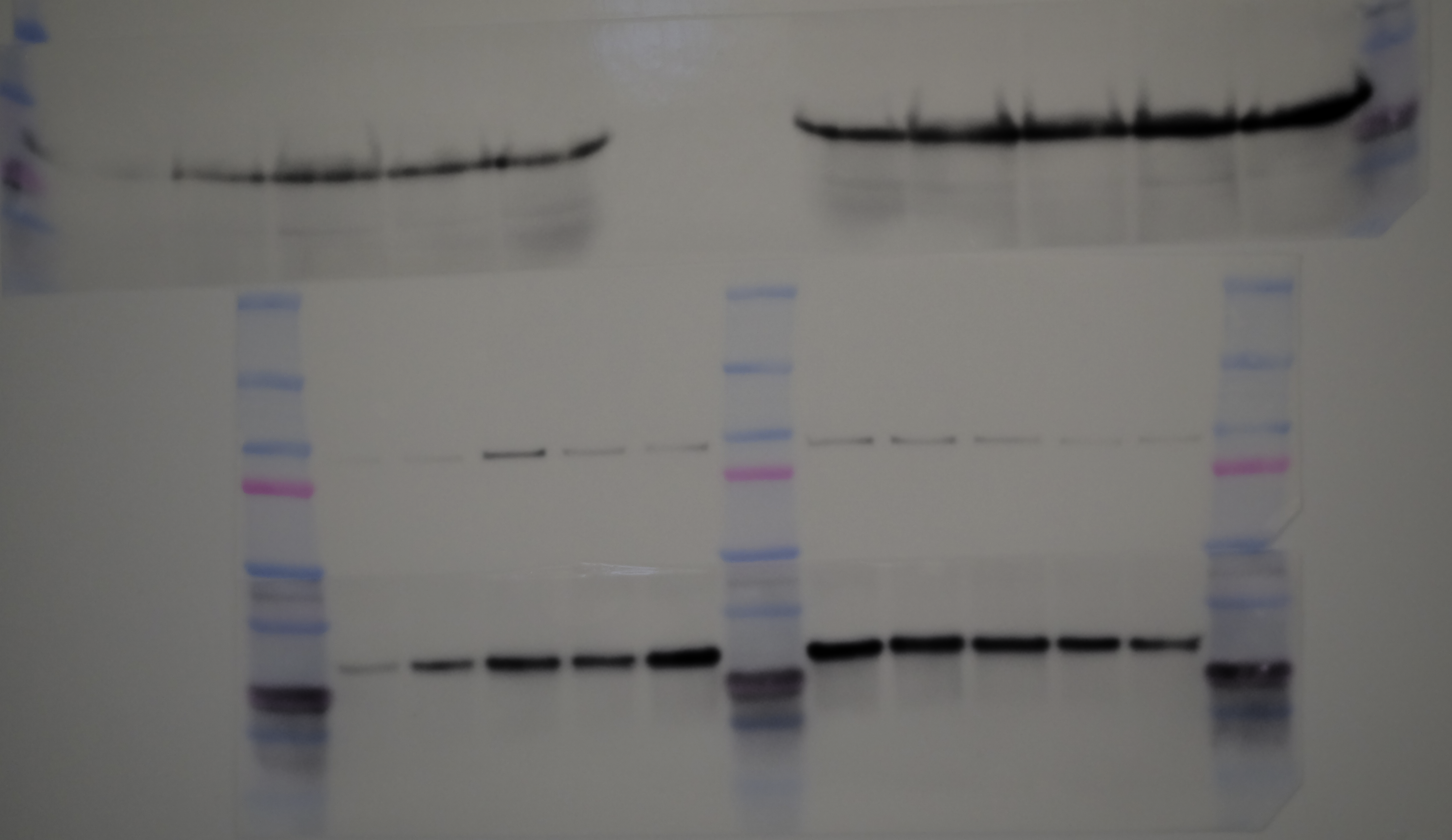

Supplement: Figure 4—source data 4. [file elife-106601-fig4-data4.zip › Figure 4-source data 4 (fig4O)/input bdh1 sno p97 sno bdh1.png]

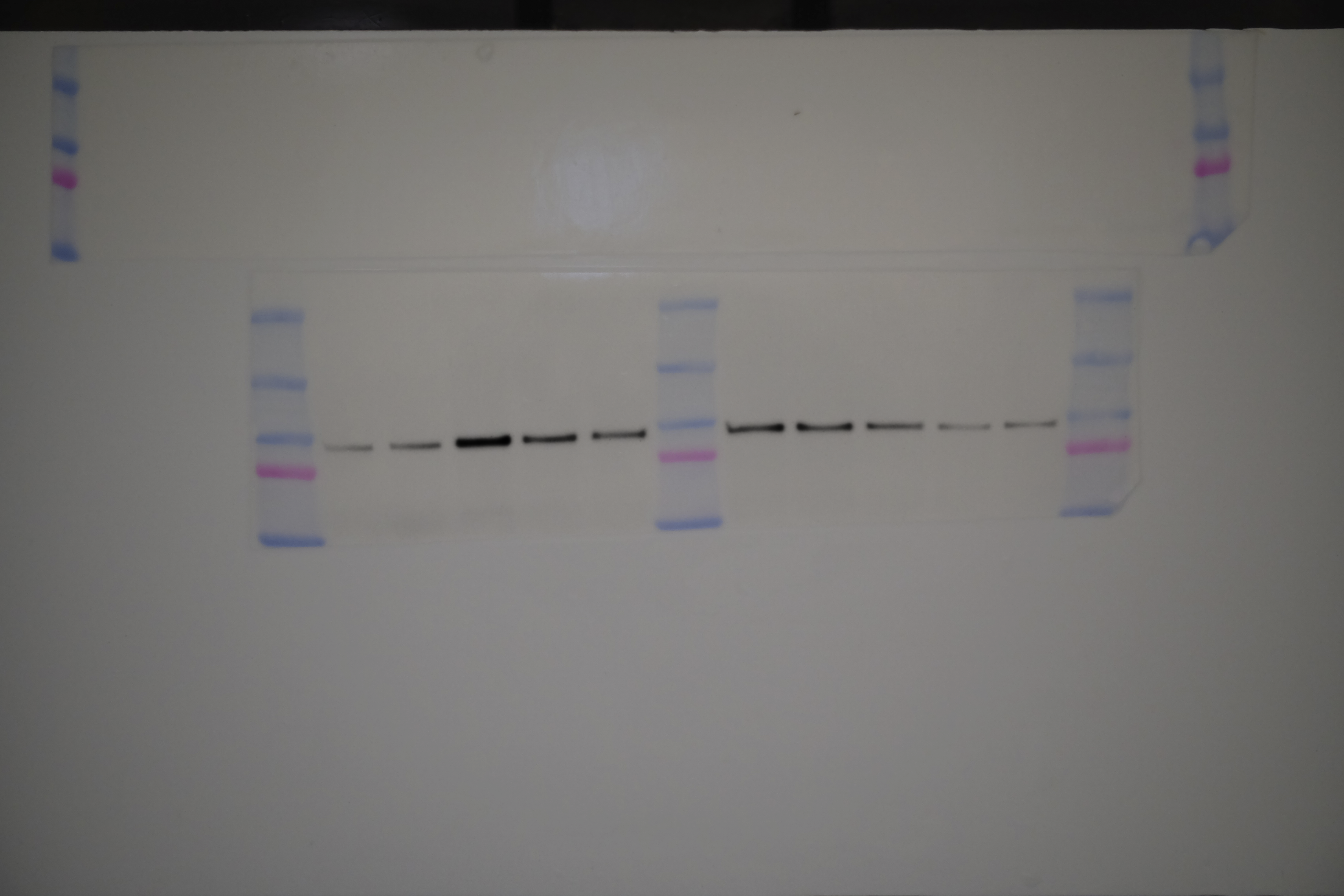

Supplement: Figure 4—source data 4. [file elife-106601-fig4-data4.zip › Figure 4-source data 4 (fig4O)/sno p97 Fig 4O.png]

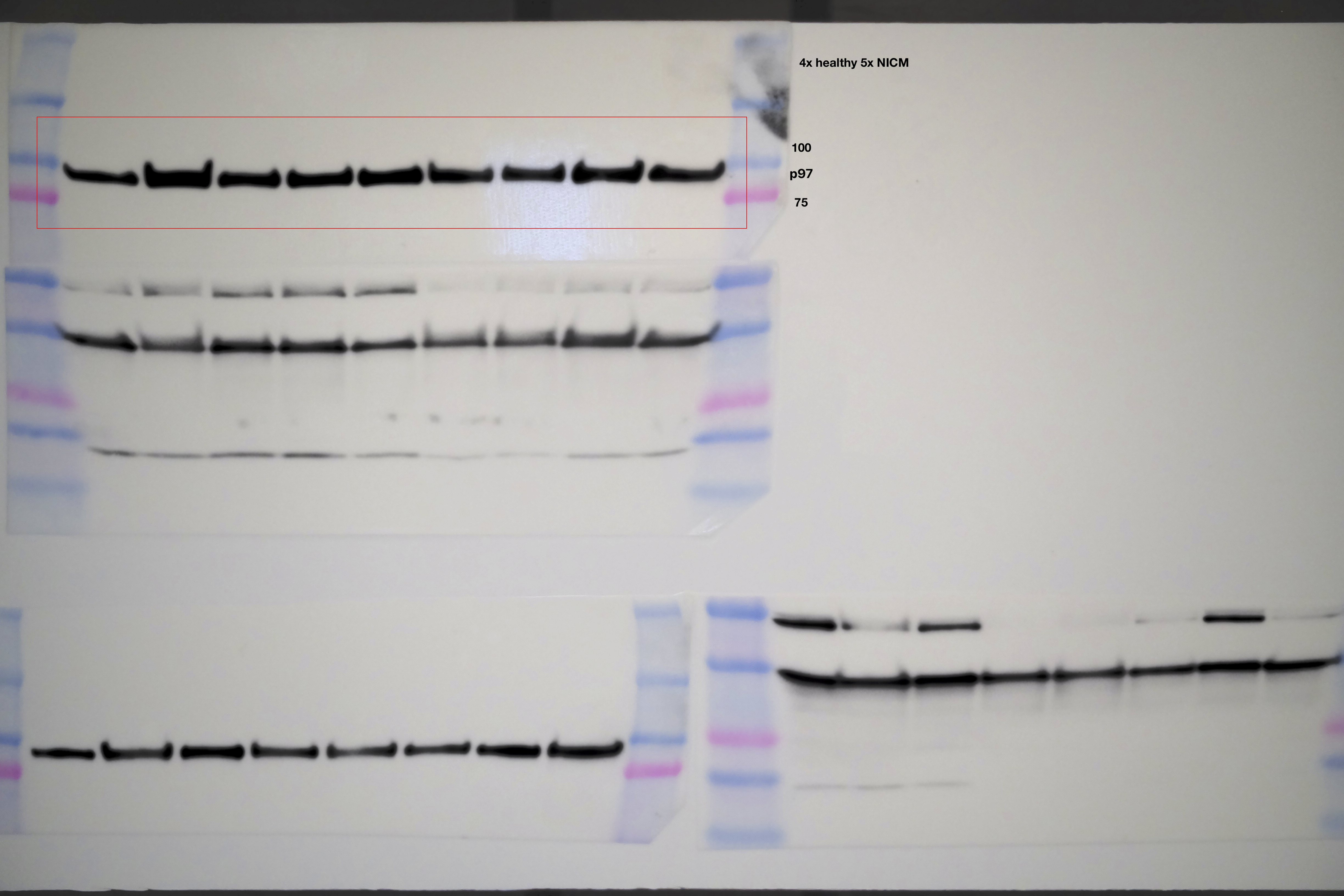

Supplement: Figure 4—source data 5. [file elife-106601-fig4-data5.zip › Figure 4-source data 5 (fig4Q)/p97 4Q.png]

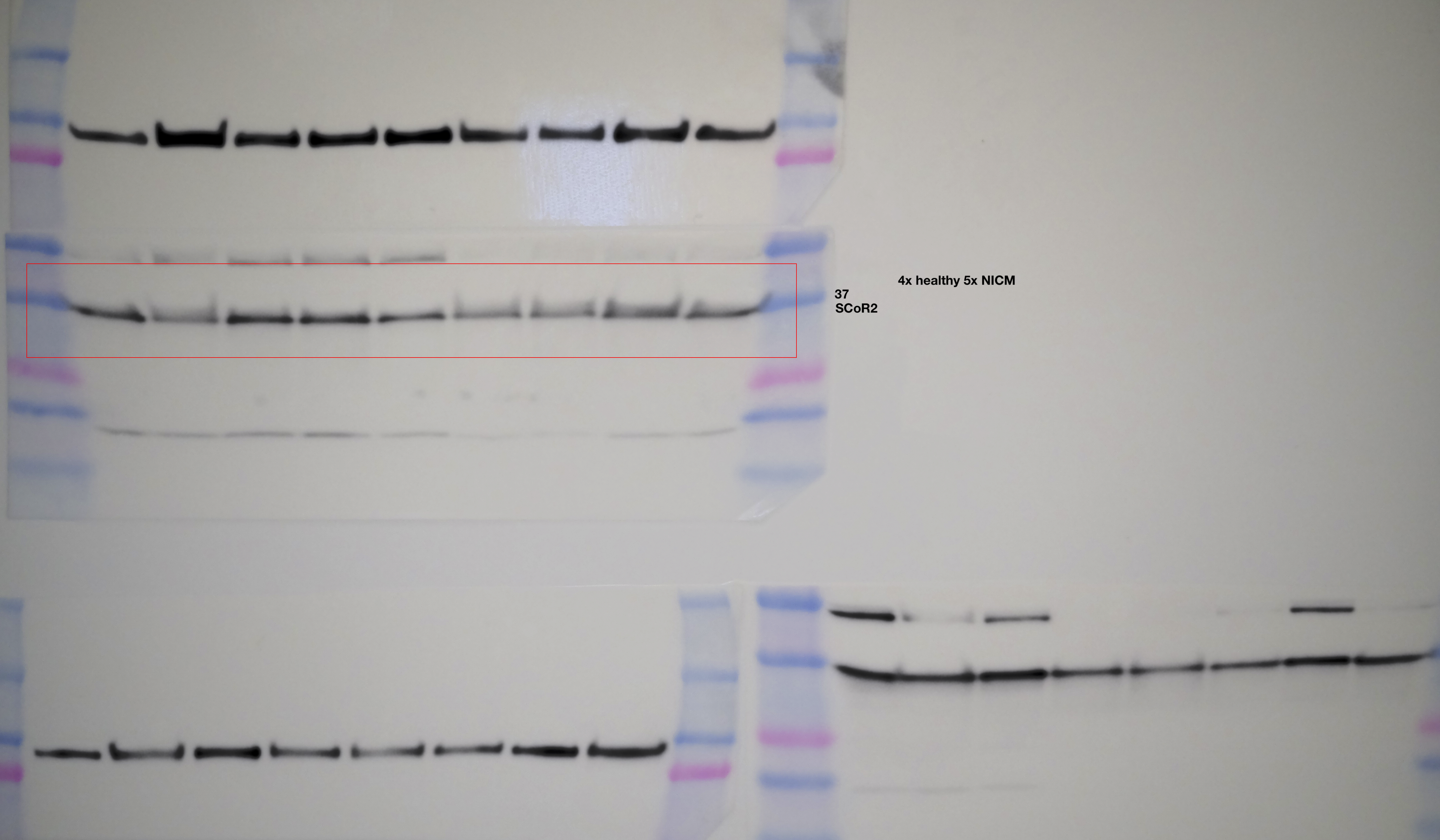

Supplement: Figure 4—source data 5. [file elife-106601-fig4-data5.zip › Figure 4-source data 5 (fig4Q)/SCoR2 4Q.png]

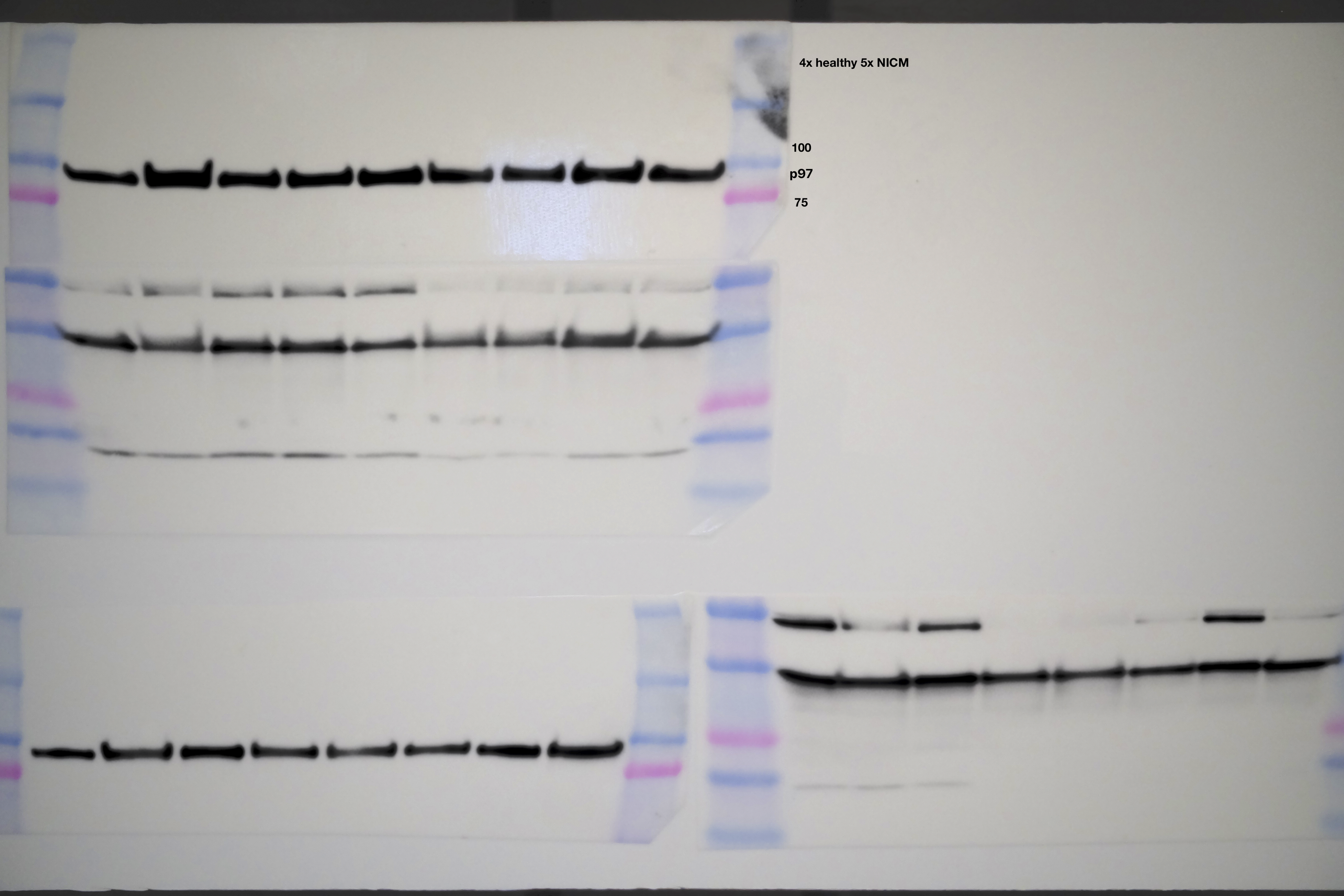

Supplement: Figure 4—source data 6. [file elife-106601-fig4-data6.zip › Figure 4-source data 6 (fig4Q)/p97 4Q.png]

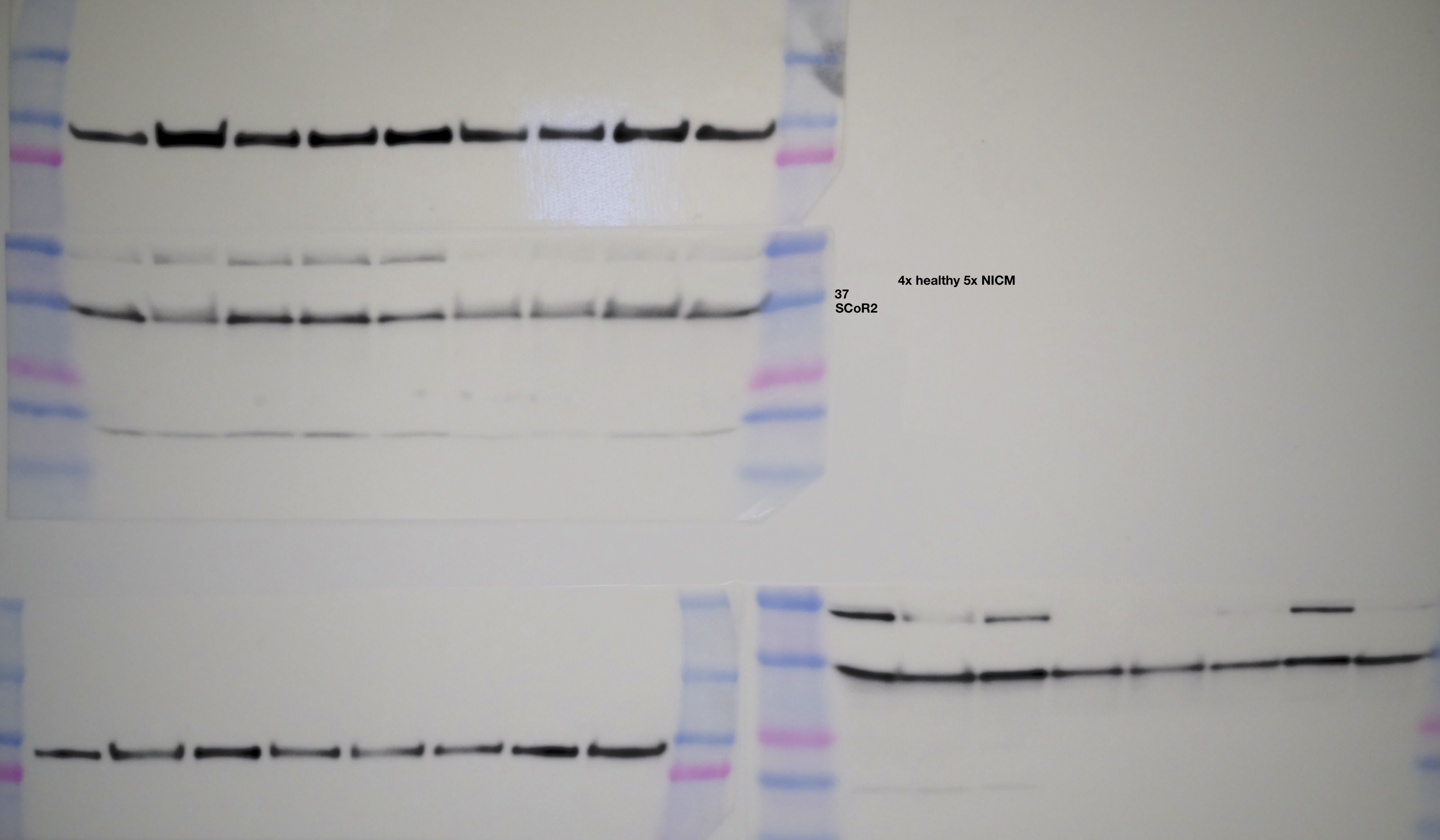

Supplement: Figure 4—source data 6. [file elife-106601-fig4-data6.zip › Figure 4-source data 6 (fig4Q)/SCoR2 4Q.png]

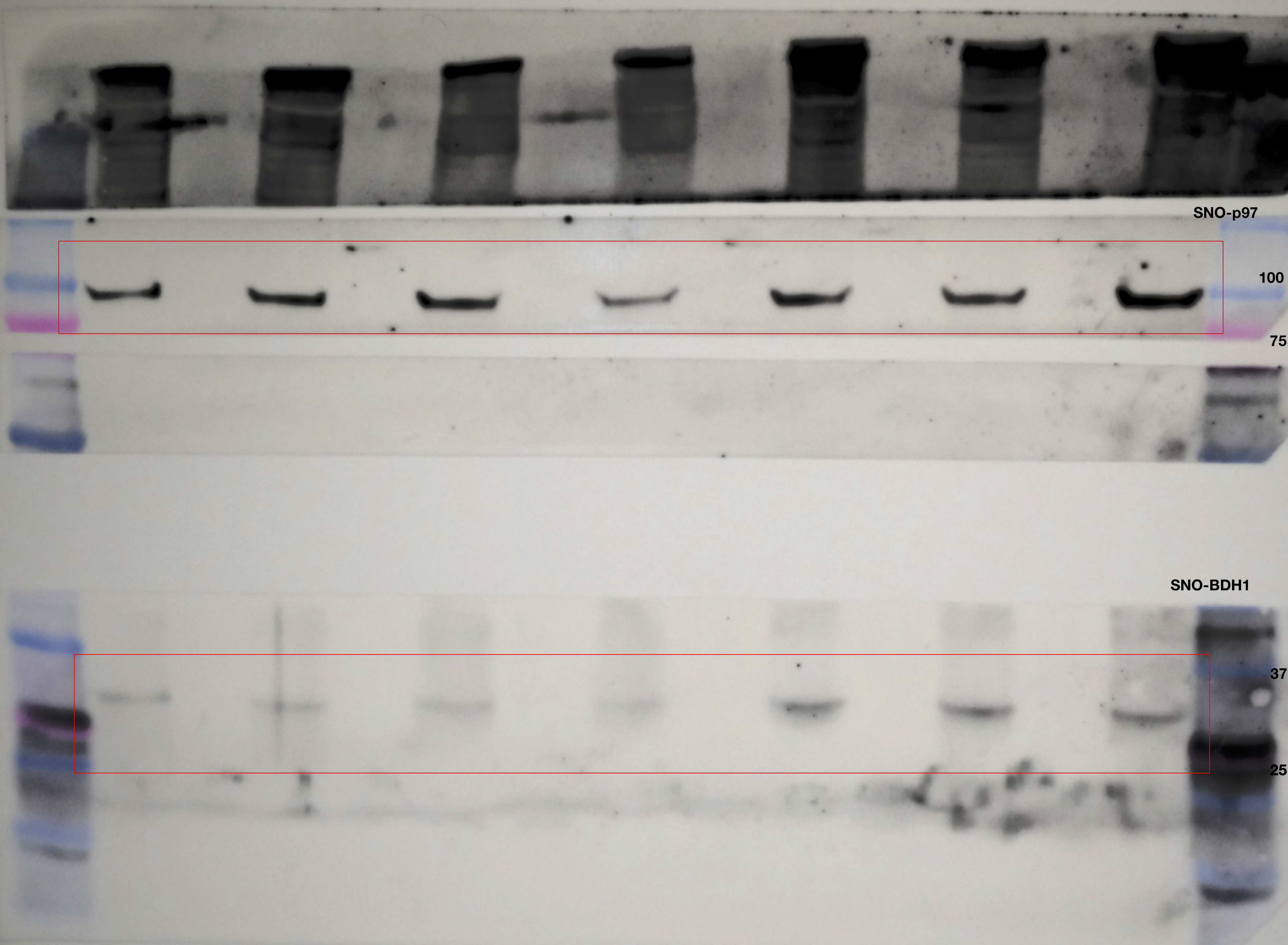

Supplement: Figure 4—figure supplement 1—source data 1. [file elife-106601-fig4-figsupp1-data1.zip › Figure 4-figure supplement 1-source data 1 (S3A)/S3A sno-bdh1 and sno-p97.png]

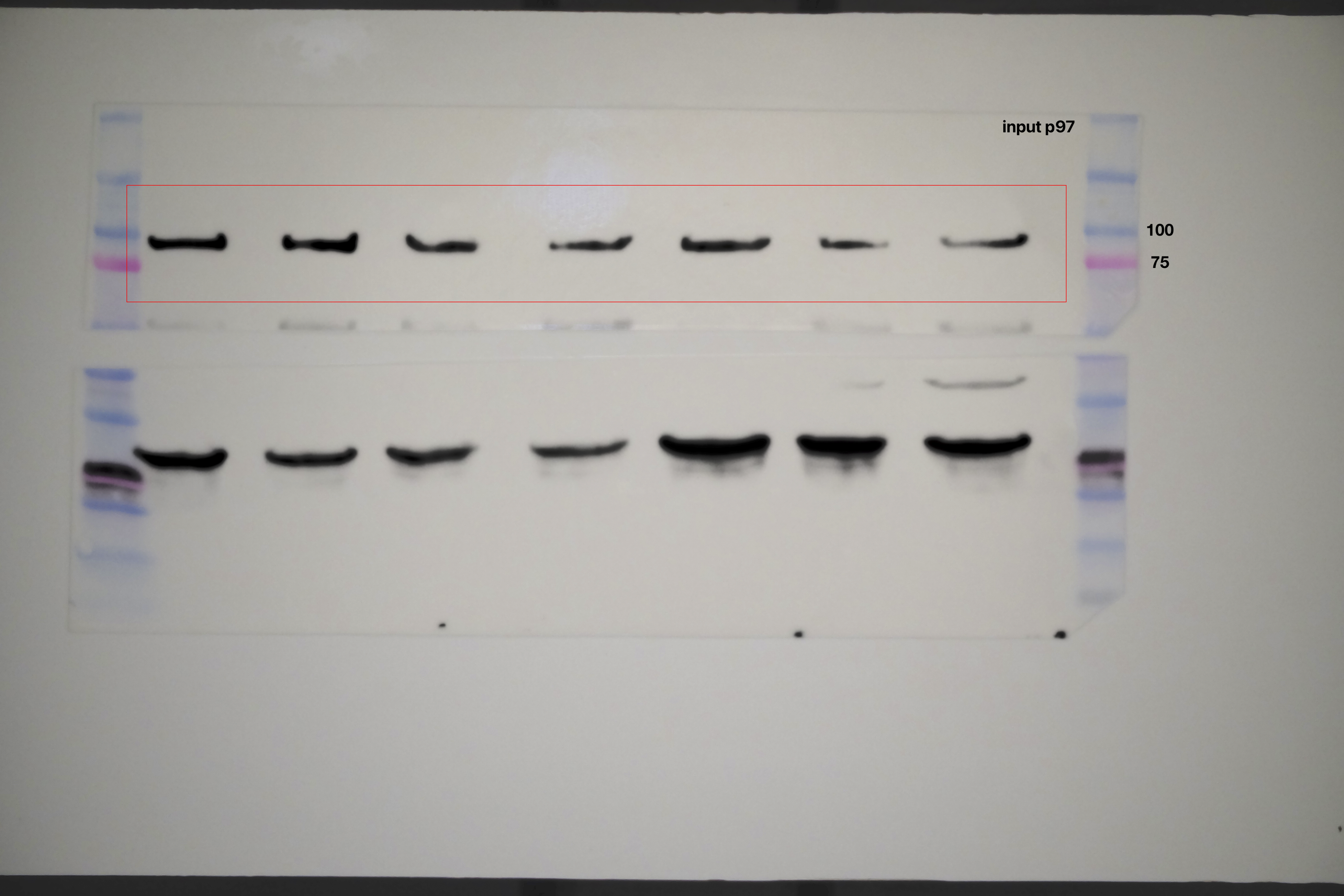

Supplement: Figure 4—figure supplement 1—source data 1. [file elife-106601-fig4-figsupp1-data1.zip › Figure 4-figure supplement 1-source data 1 (S3A)/S3A p97 input.png]

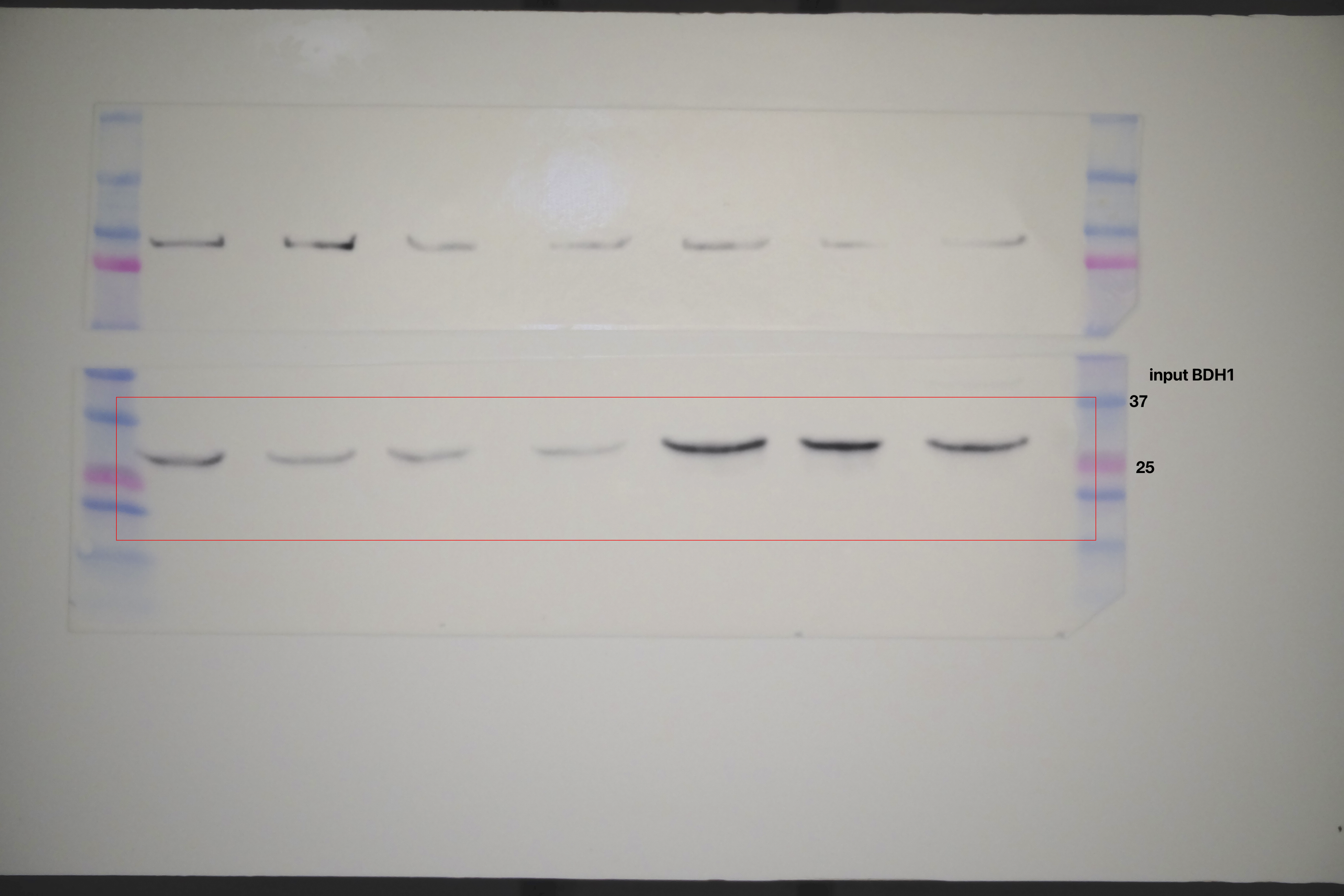

Supplement: Figure 4—figure supplement 1—source data 1. [file elife-106601-fig4-figsupp1-data1.zip › Figure 4-figure supplement 1-source data 1 (S3A)/S3A BDH1 input.png]

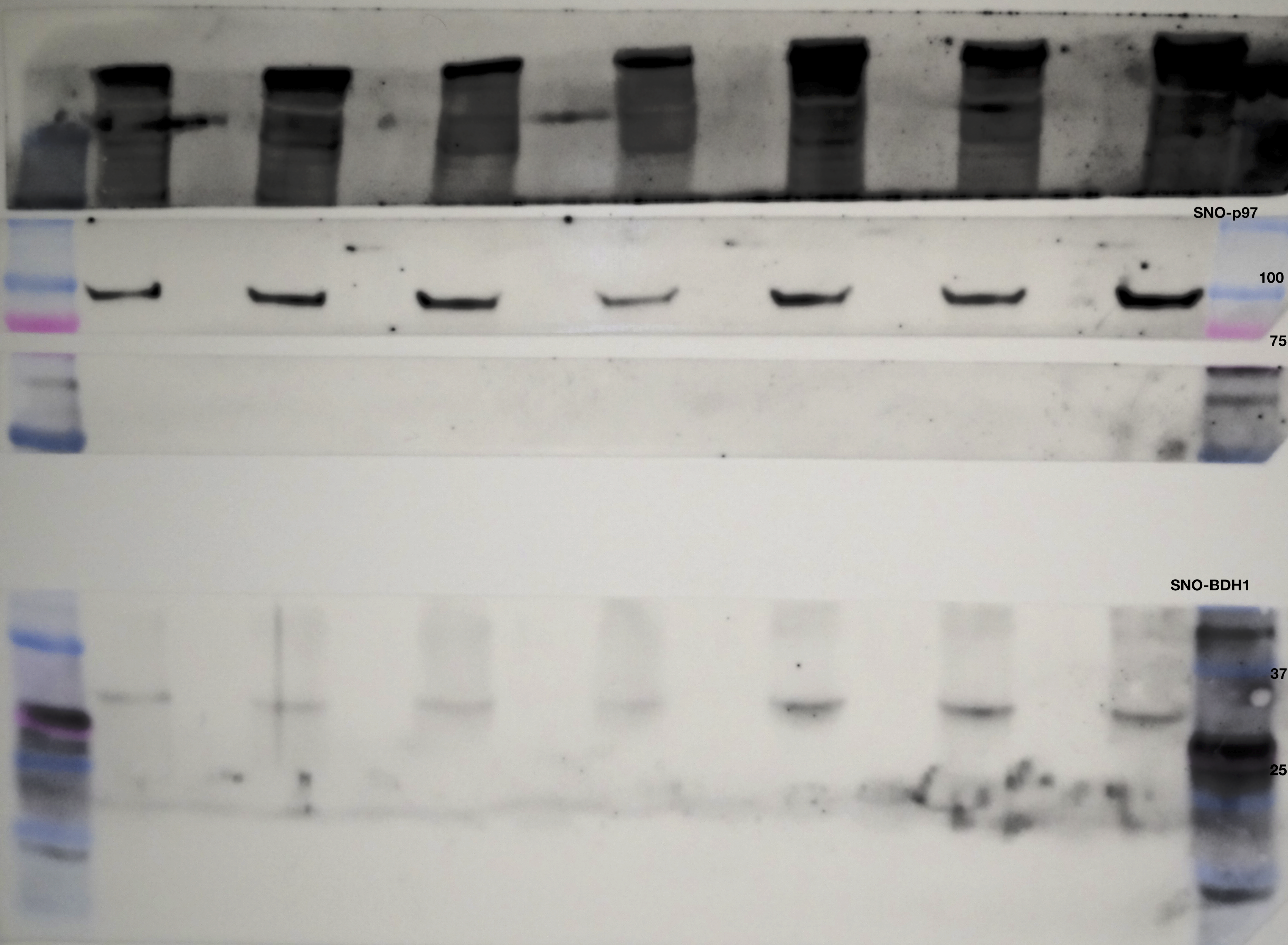

Supplement: Figure 4—figure supplement 1—source data 2. [file elife-106601-fig4-figsupp1-data2.zip › Figure 4-figure supplement 1-source data 2 (S3A)/sno-bdh1 and sno-p97.png]

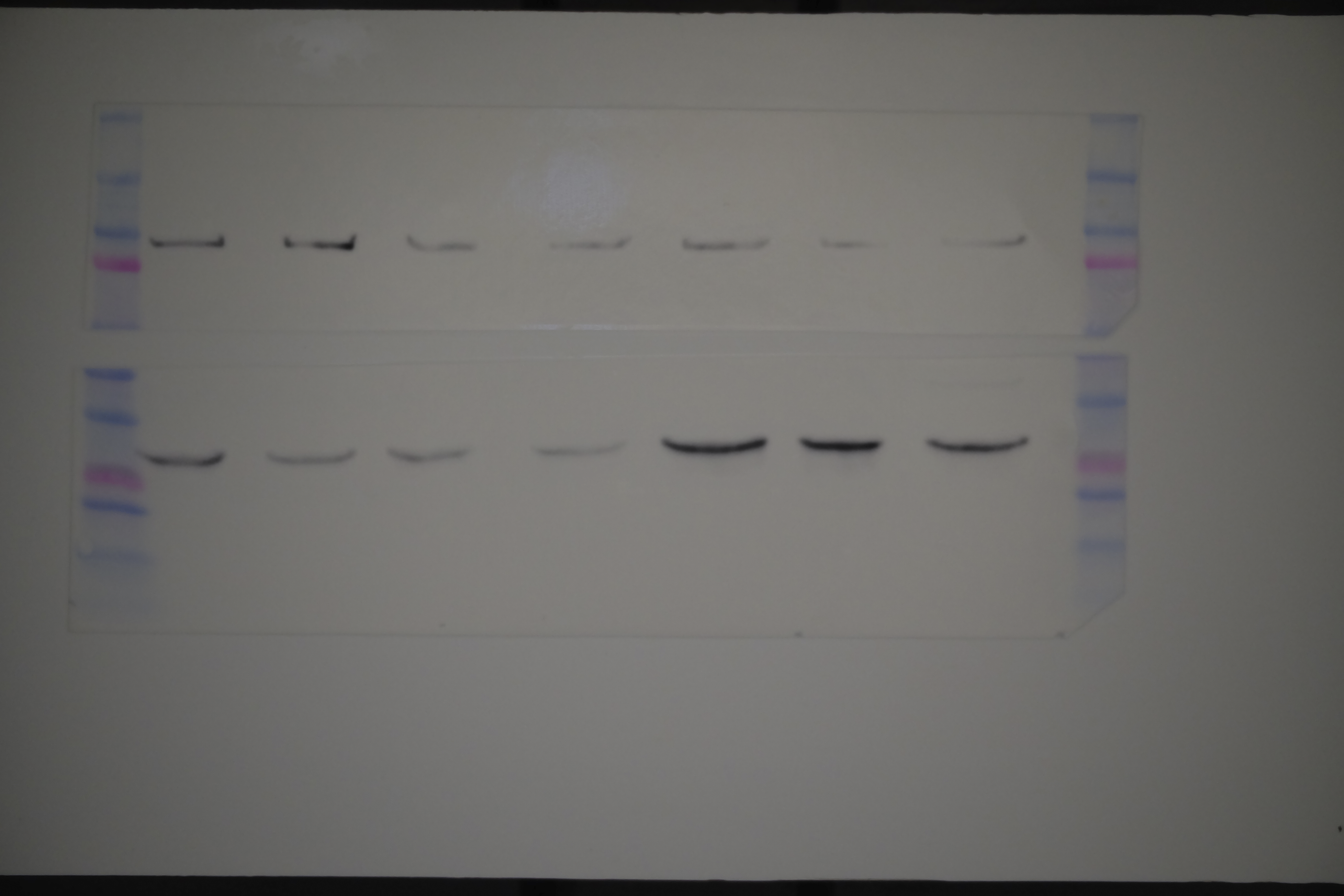

Supplement: Figure 4—figure supplement 1—source data 2. [file elife-106601-fig4-figsupp1-data2.zip › Figure 4-figure supplement 1-source data 2 (S3A)/BDH1 input.png]

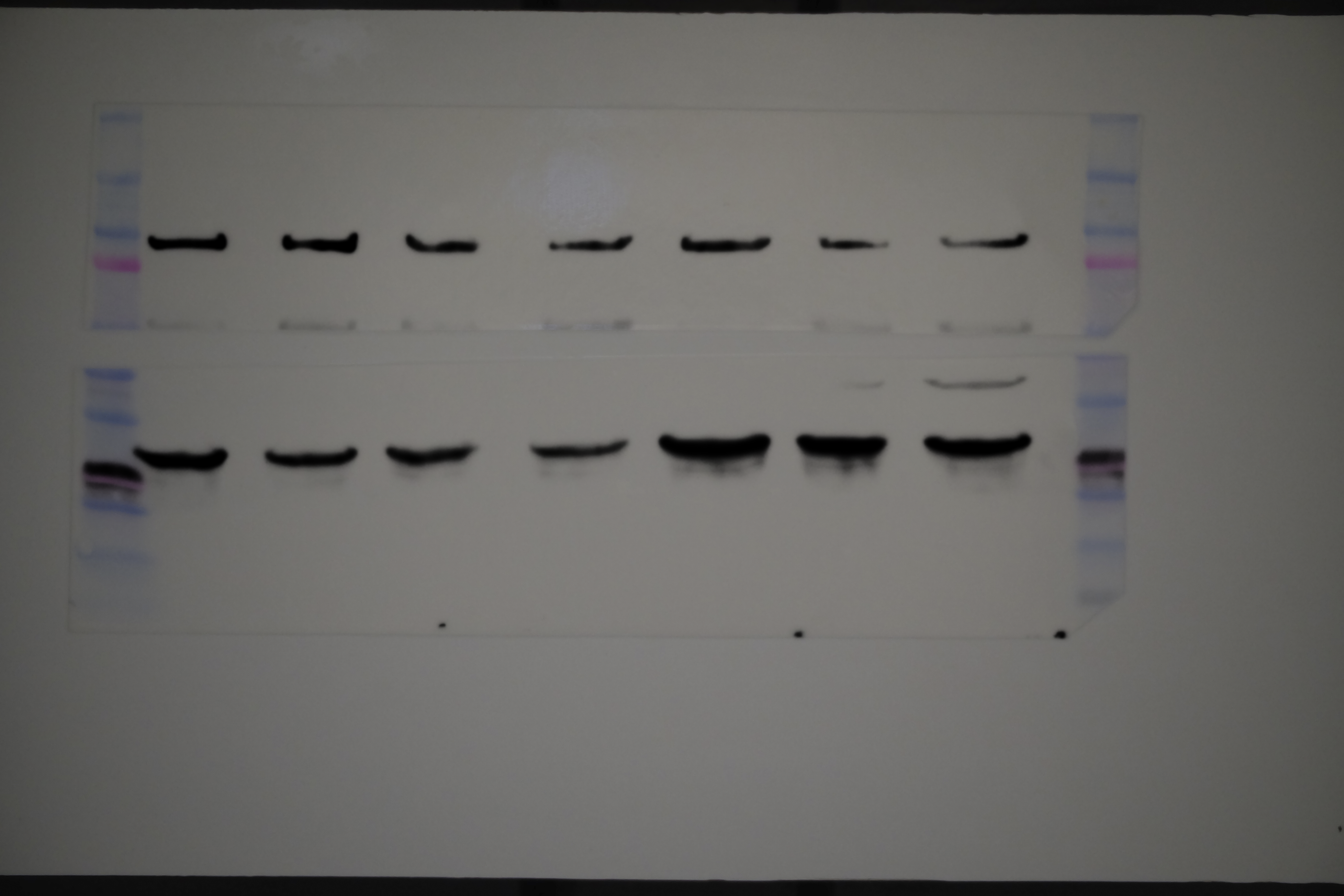

Supplement: Figure 4—figure supplement 1—source data 2. [file elife-106601-fig4-figsupp1-data2.zip › Figure 4-figure supplement 1-source data 2 (S3A)/p97 input.png]

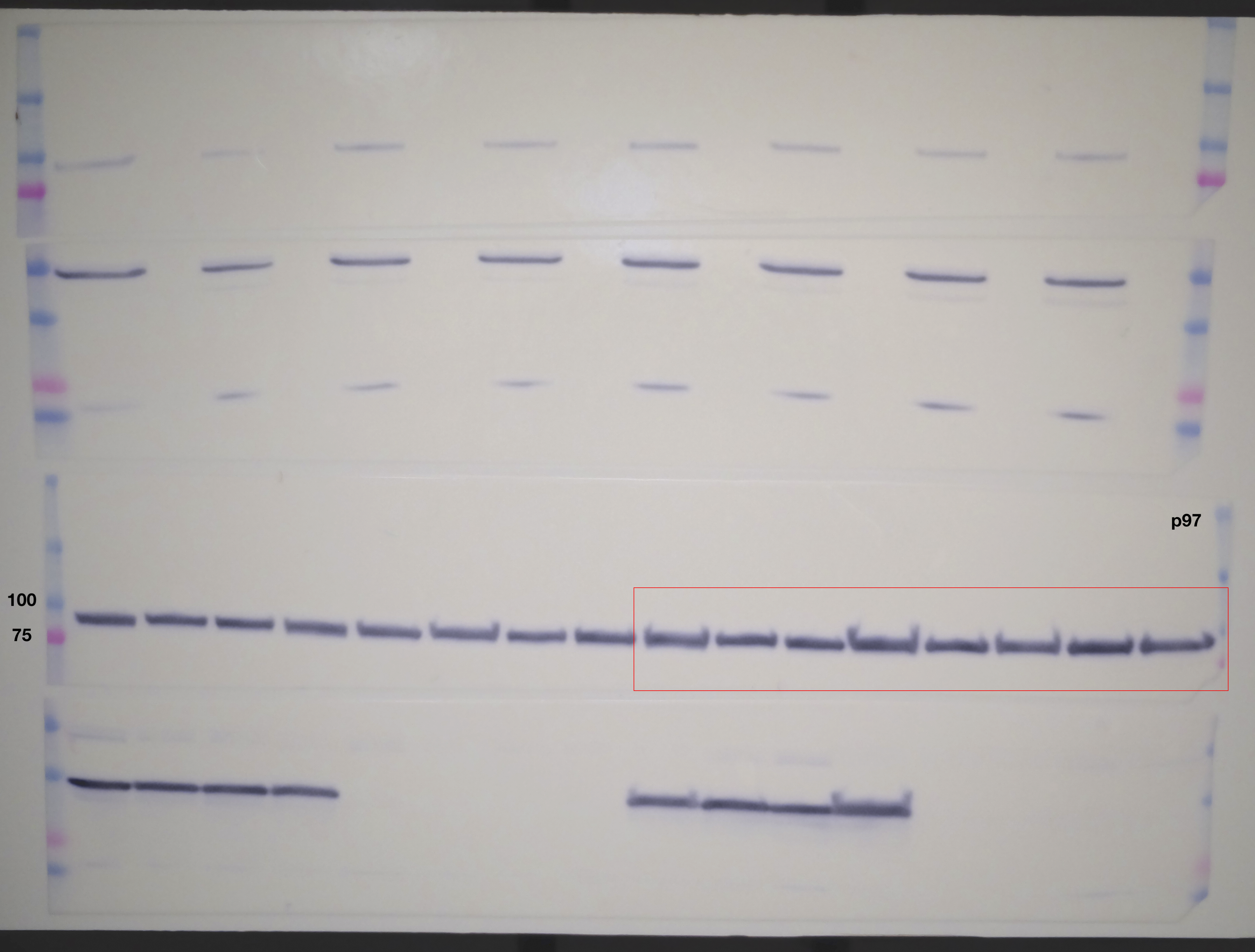

Supplement: Figure 4—figure supplement 1—source data 3. [file elife-106601-fig4-figsupp1-data3.zip › Figure 4-figure supplement 1-source data 3 (S3B)/S3B p97 input.png]

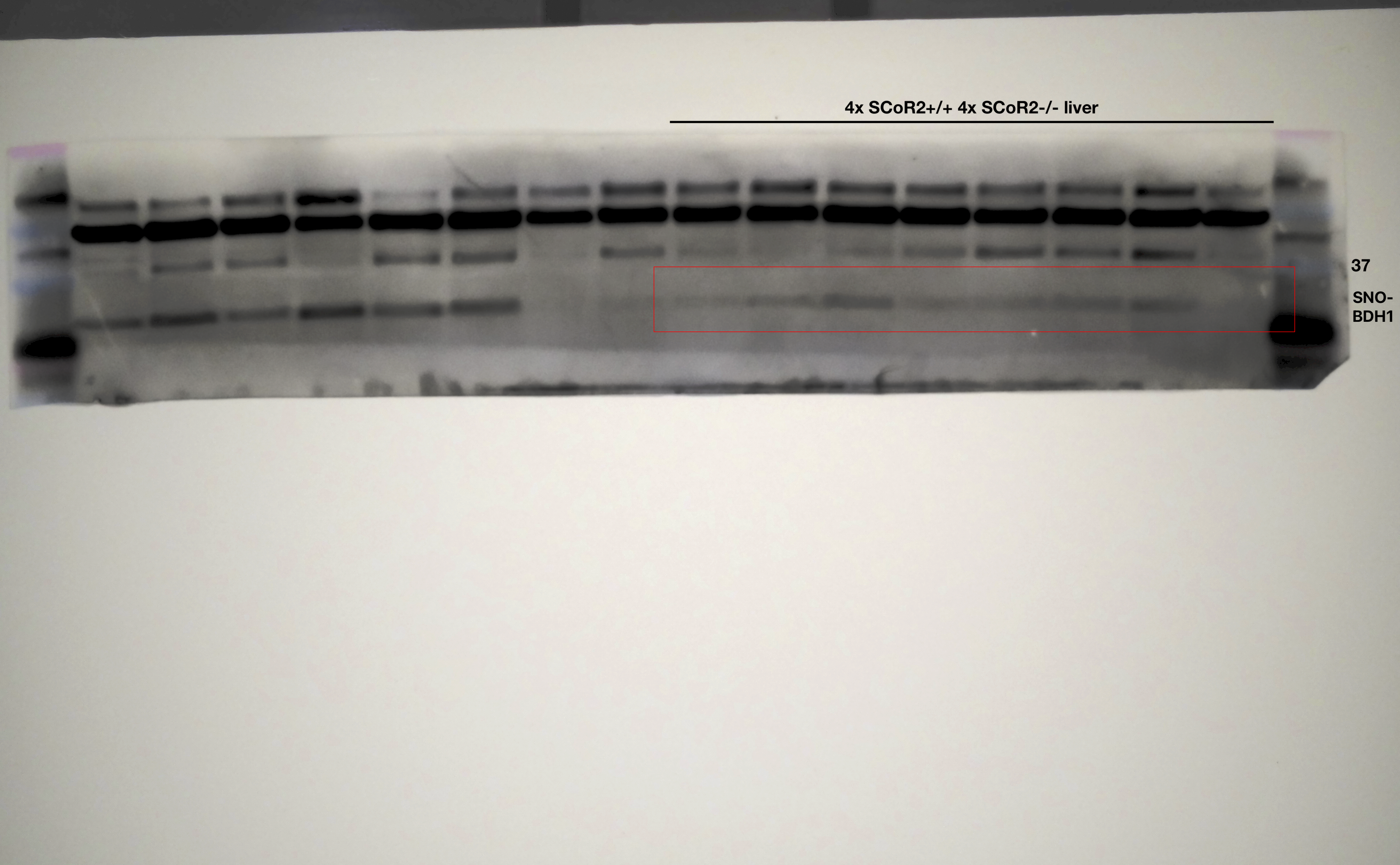

Supplement: Figure 4—figure supplement 1—source data 3. [file elife-106601-fig4-figsupp1-data3.zip › Figure 4-figure supplement 1-source data 3 (S3B)/SNO-BDH1 S3B.png]

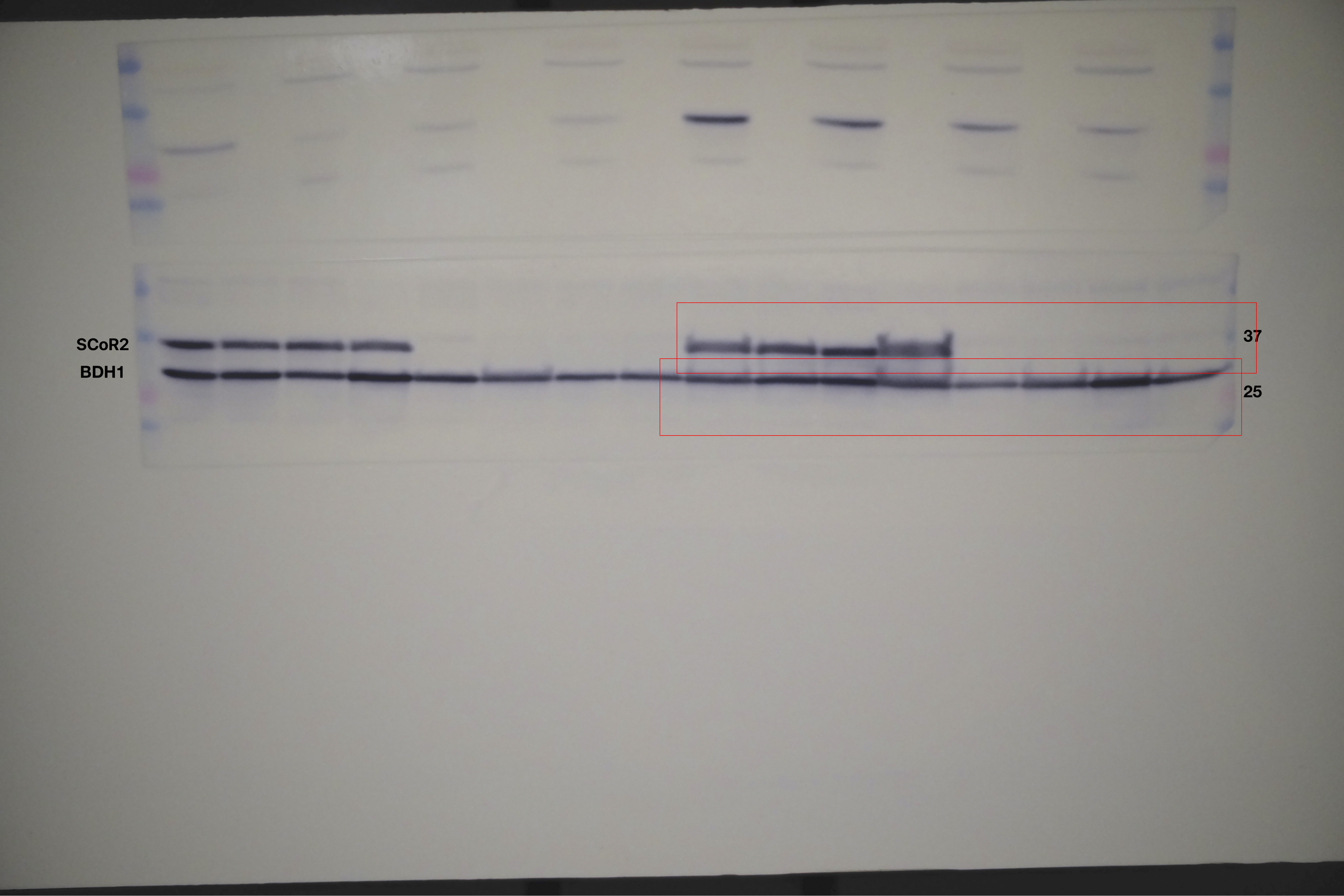

Supplement: Figure 4—figure supplement 1—source data 3. [file elife-106601-fig4-figsupp1-data3.zip › Figure 4-figure supplement 1-source data 3 (S3B)/S3B SCoR2 and BDH1 input.png]

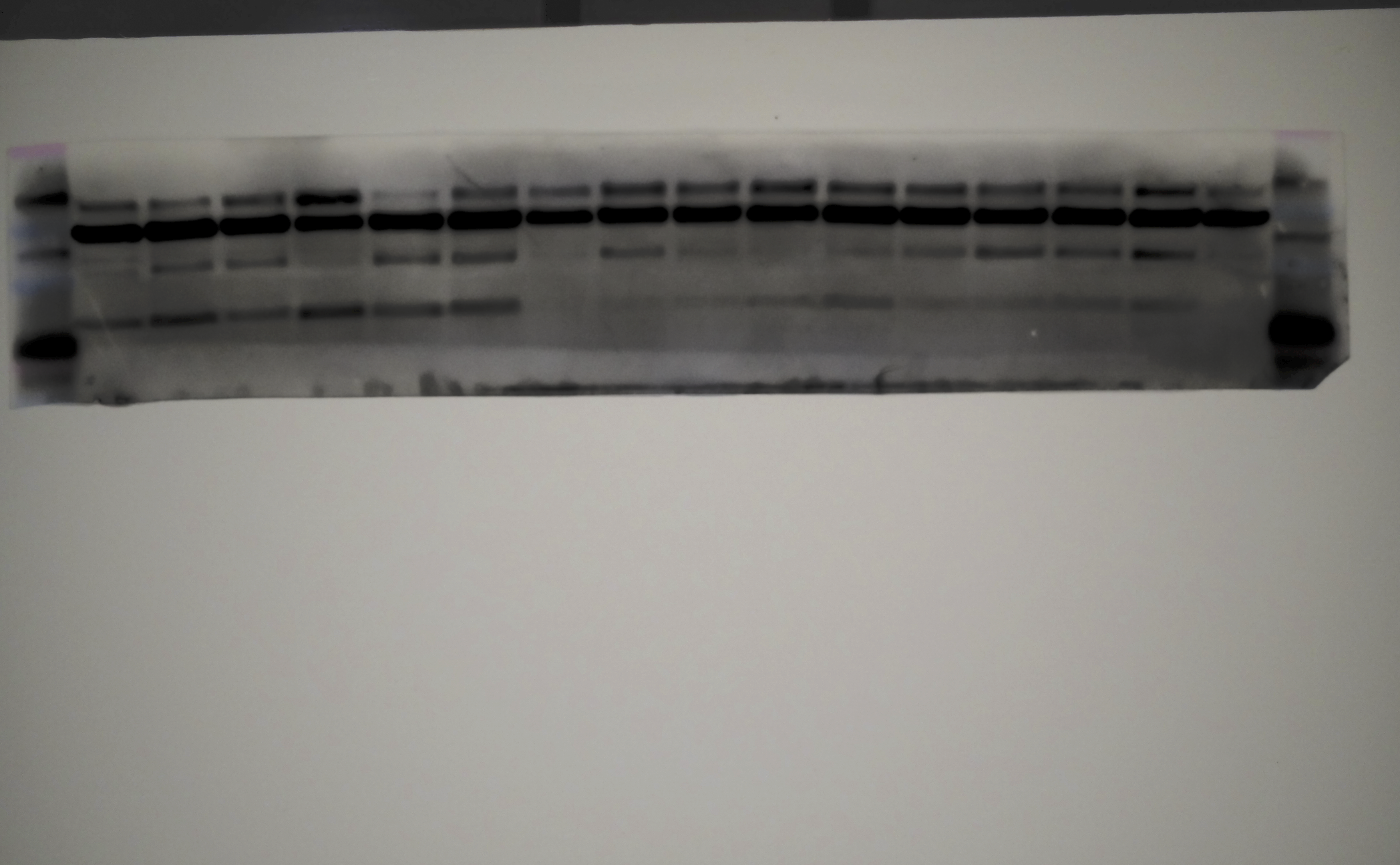

Supplement: Figure 4—figure supplement 1—source data 4. [file elife-106601-fig4-figsupp1-data4.zip › Figure 4-figure supplement 1-source data 4 (S3B)/SNO-BDH1.png]

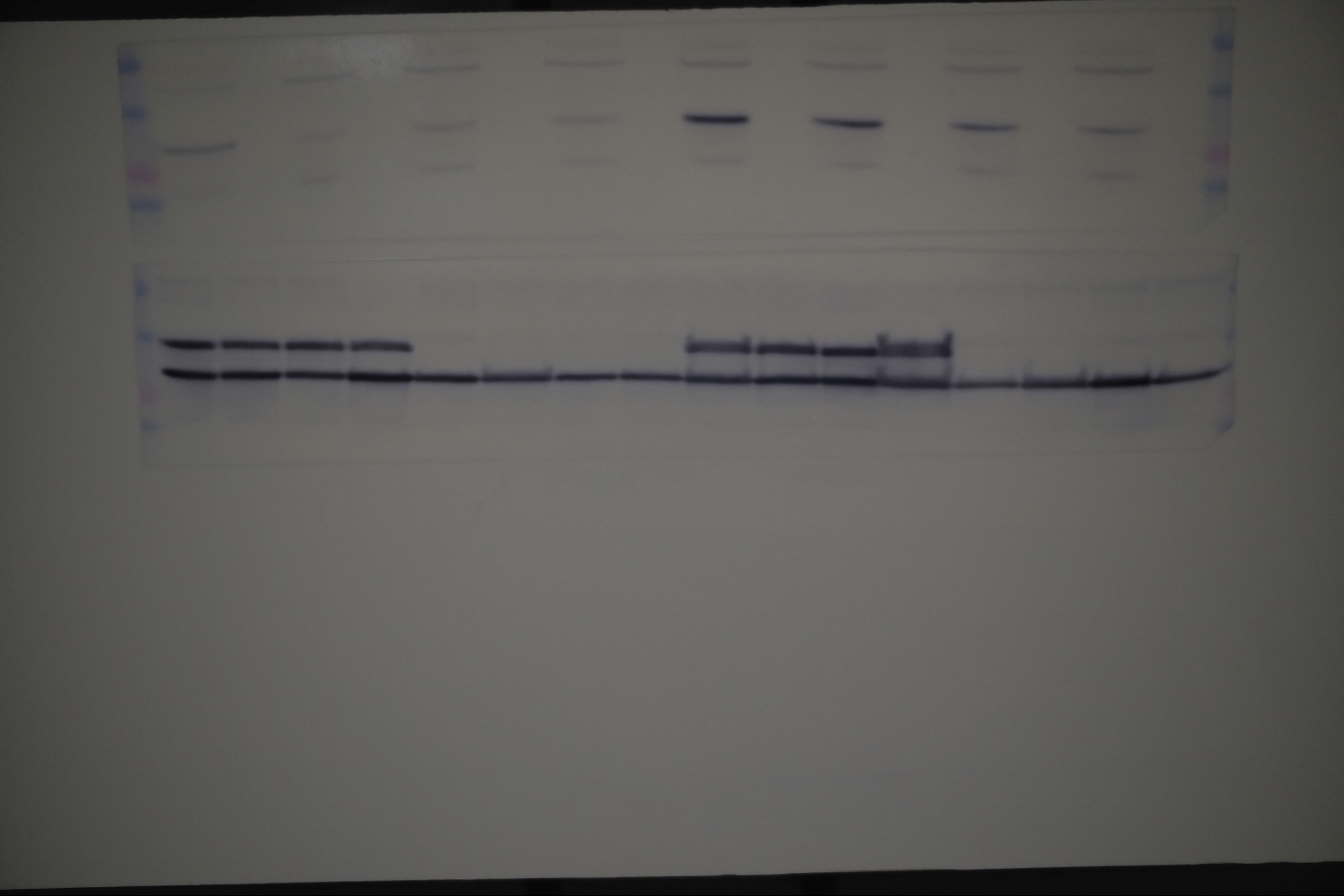

Supplement: Figure 4—figure supplement 1—source data 4. [file elife-106601-fig4-figsupp1-data4.zip › Figure 4-figure supplement 1-source data 4 (S3B)/input SCoR2 and BDH1 .png]

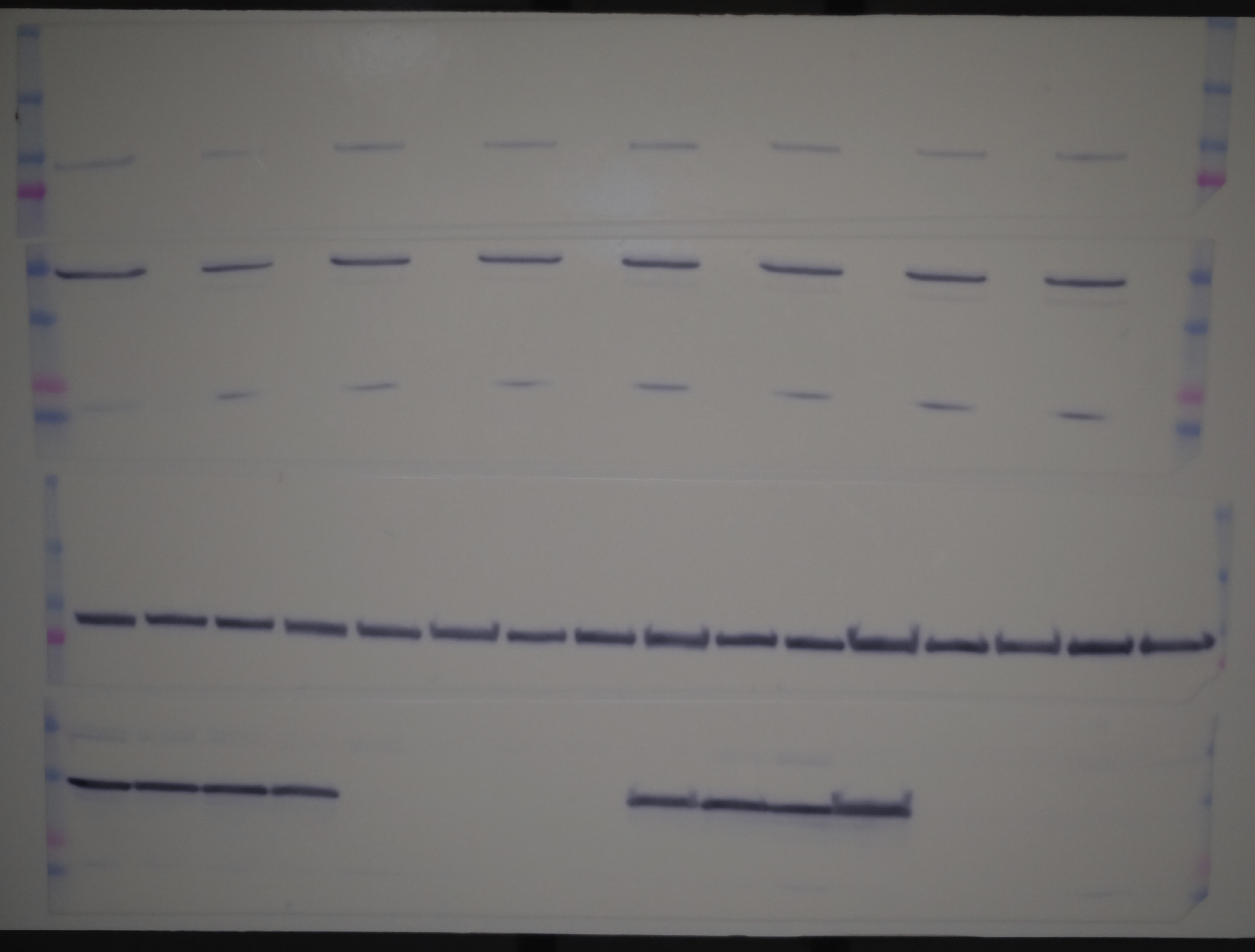

Supplement: Figure 4—figure supplement 1—source data 4. [file elife-106601-fig4-figsupp1-data4.zip › Figure 4-figure supplement 1-source data 4 (S3B)/input p97.png]

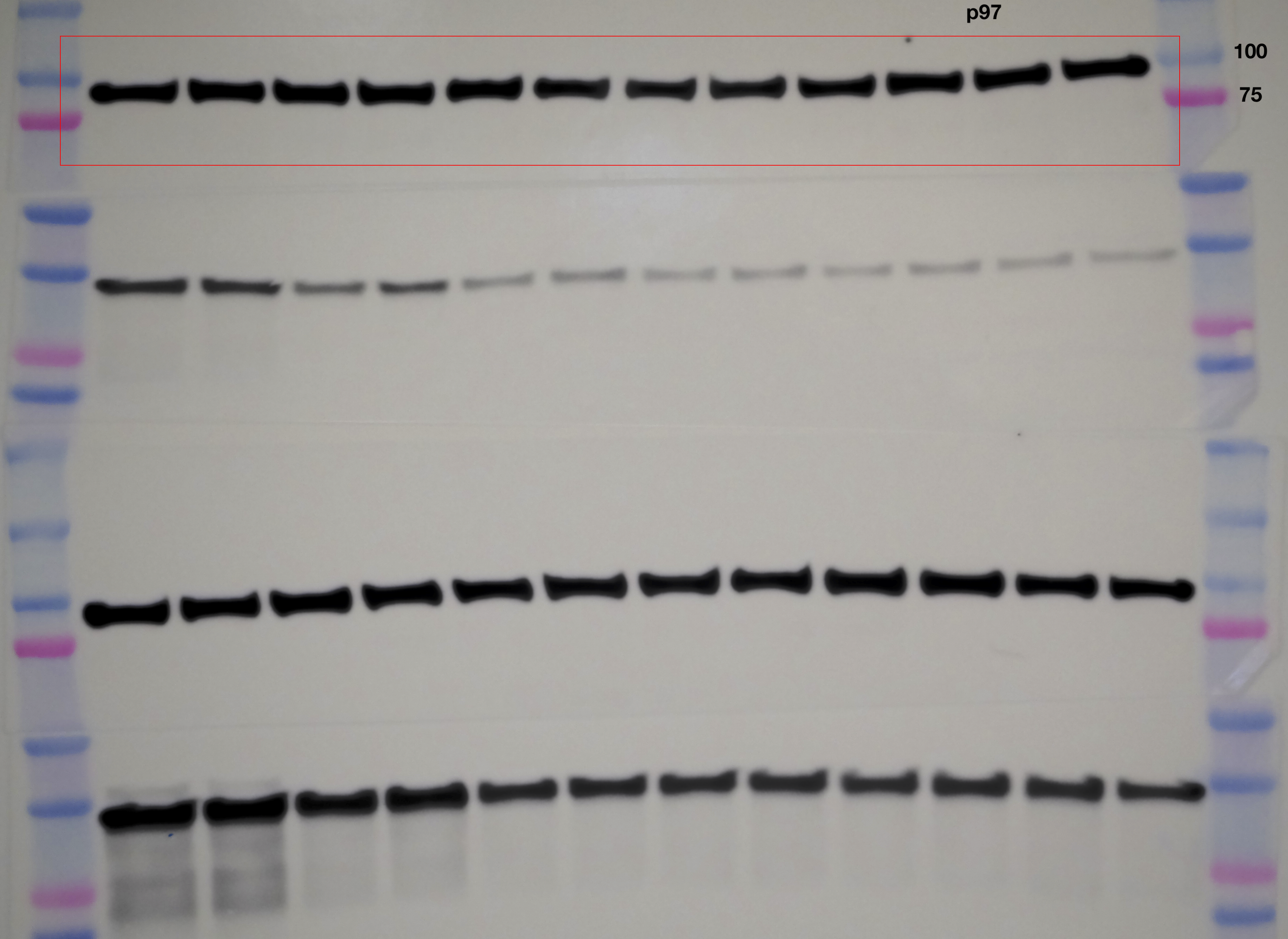

Supplement: Figure 4—figure supplement 1—source data 5. [file elife-106601-fig4-figsupp1-data5.zip › Figure 4-figure supplement 1-source data 5 (S3C)/S3C WT + veh (p97).png]

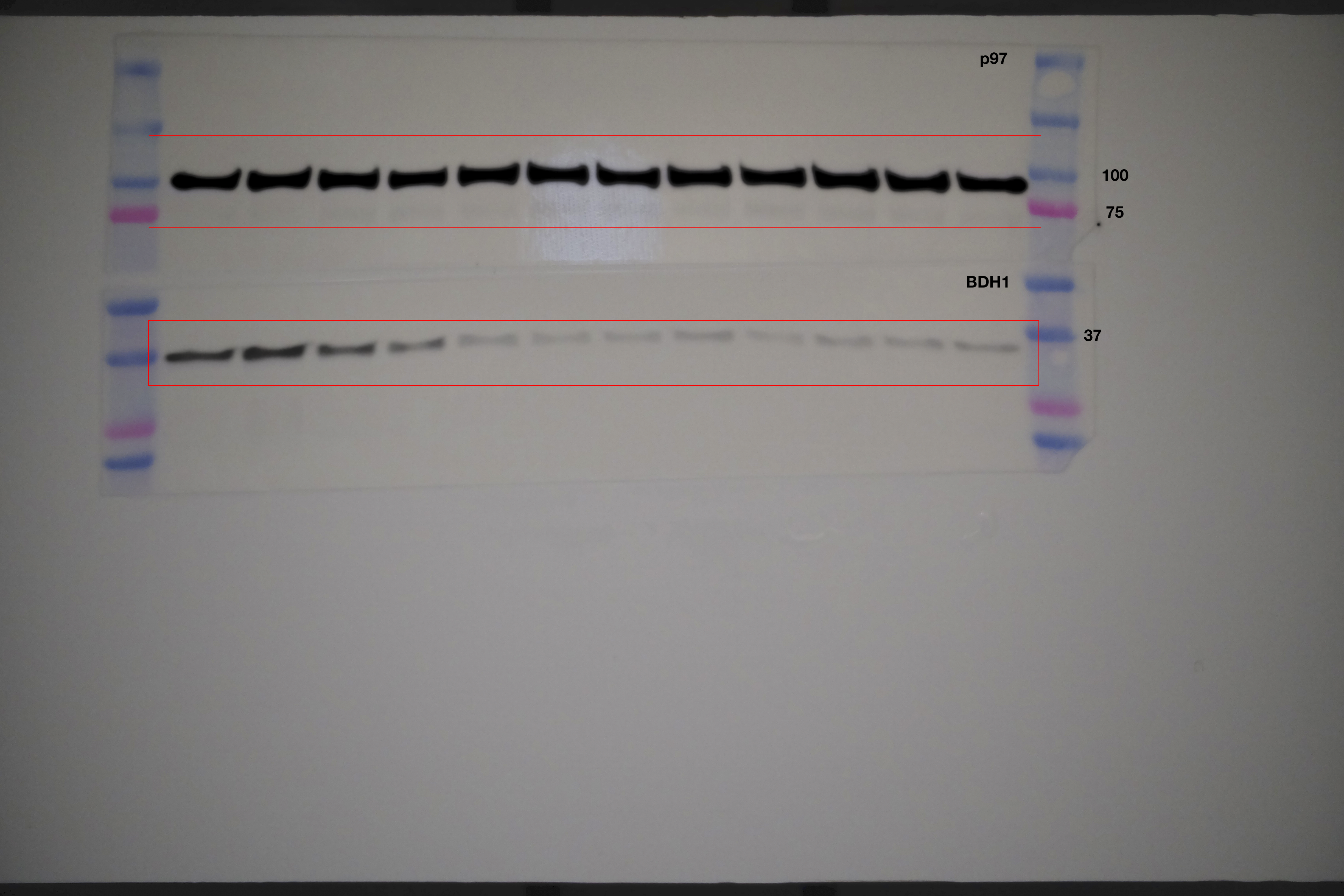

Supplement: Figure 4—figure supplement 1—source data 5. [file elife-106601-fig4-figsupp1-data5.zip › Figure 4-figure supplement 1-source data 5 (S3C)/S3C C115S BDH1 + ECNO.png]

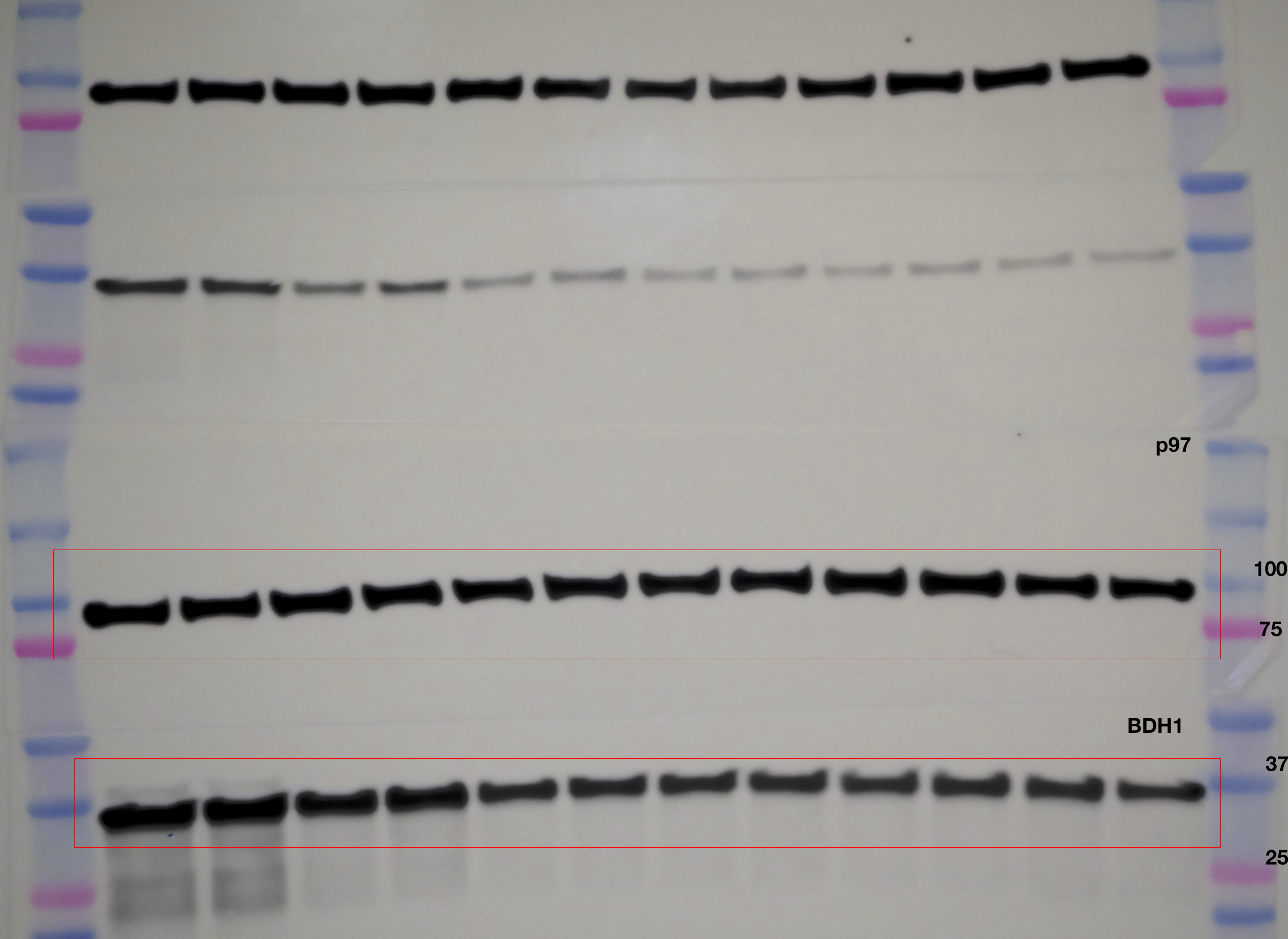

Supplement: Figure 4—figure supplement 1—source data 5. [file elife-106601-fig4-figsupp1-data5.zip › Figure 4-figure supplement 1-source data 5 (S3C)/S3C WT + ECNO (p97 and BDH1).png]

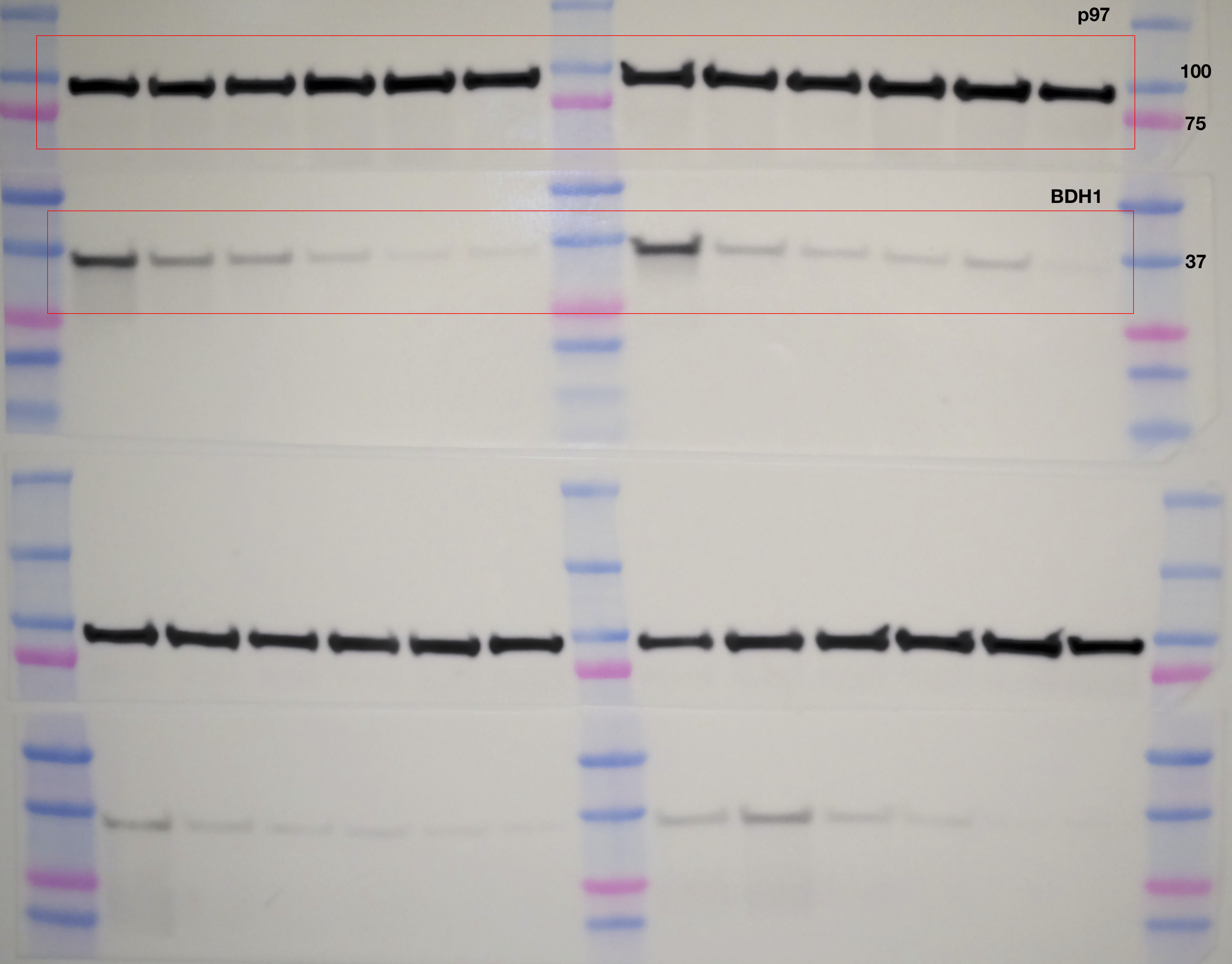

Supplement: Figure 4—figure supplement 1—source data 5. [file elife-106601-fig4-figsupp1-data5.zip › Figure 4-figure supplement 1-source data 5 (S3C)/S3C C115S BDH1 + veh (p97 and BDH1).png]

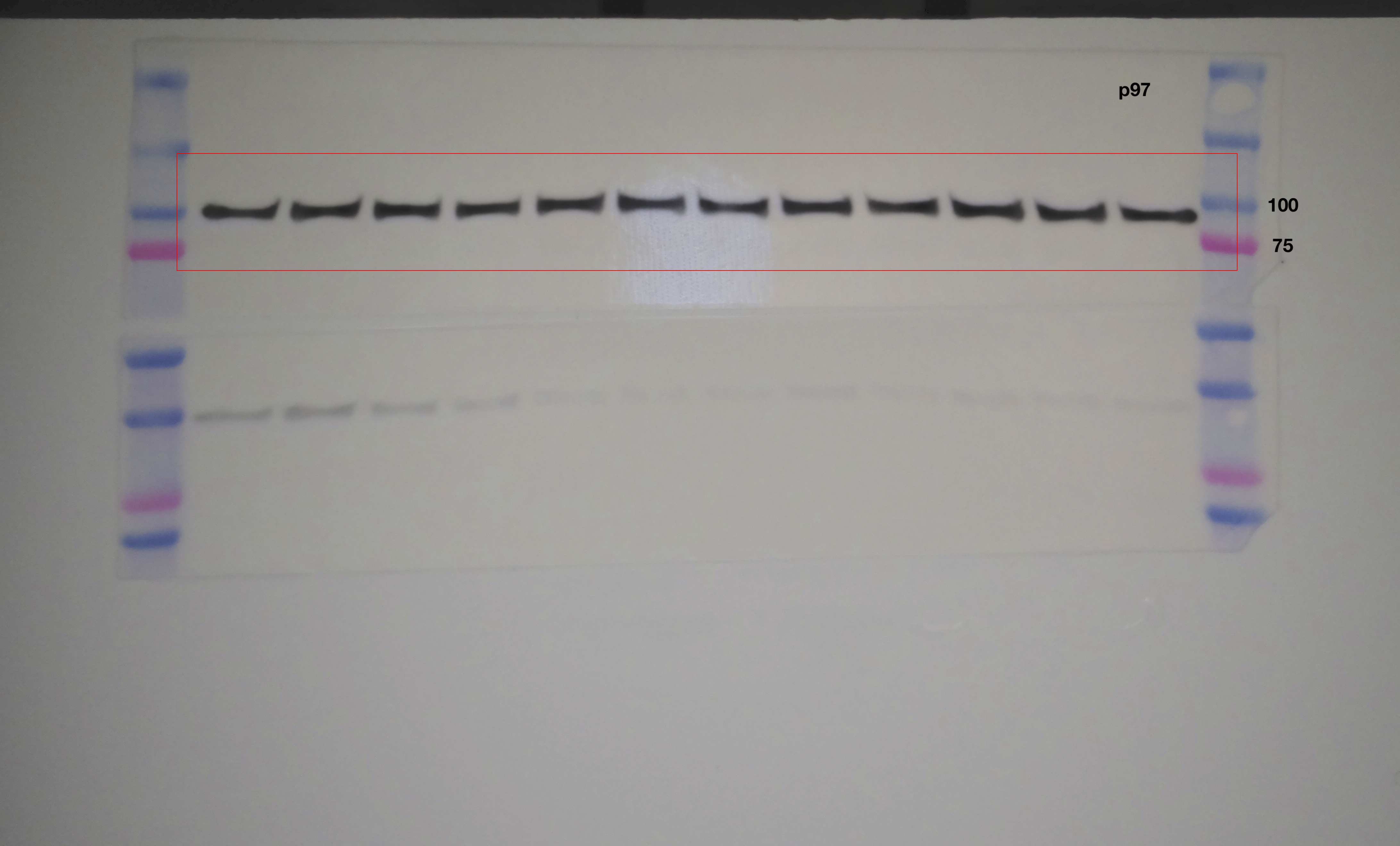

Supplement: Figure 4—figure supplement 1—source data 5. [file elife-106601-fig4-figsupp1-data5.zip › Figure 4-figure supplement 1-source data 5 (S3C)/S3C C115S ECNO p97 (just p97, on top).png]

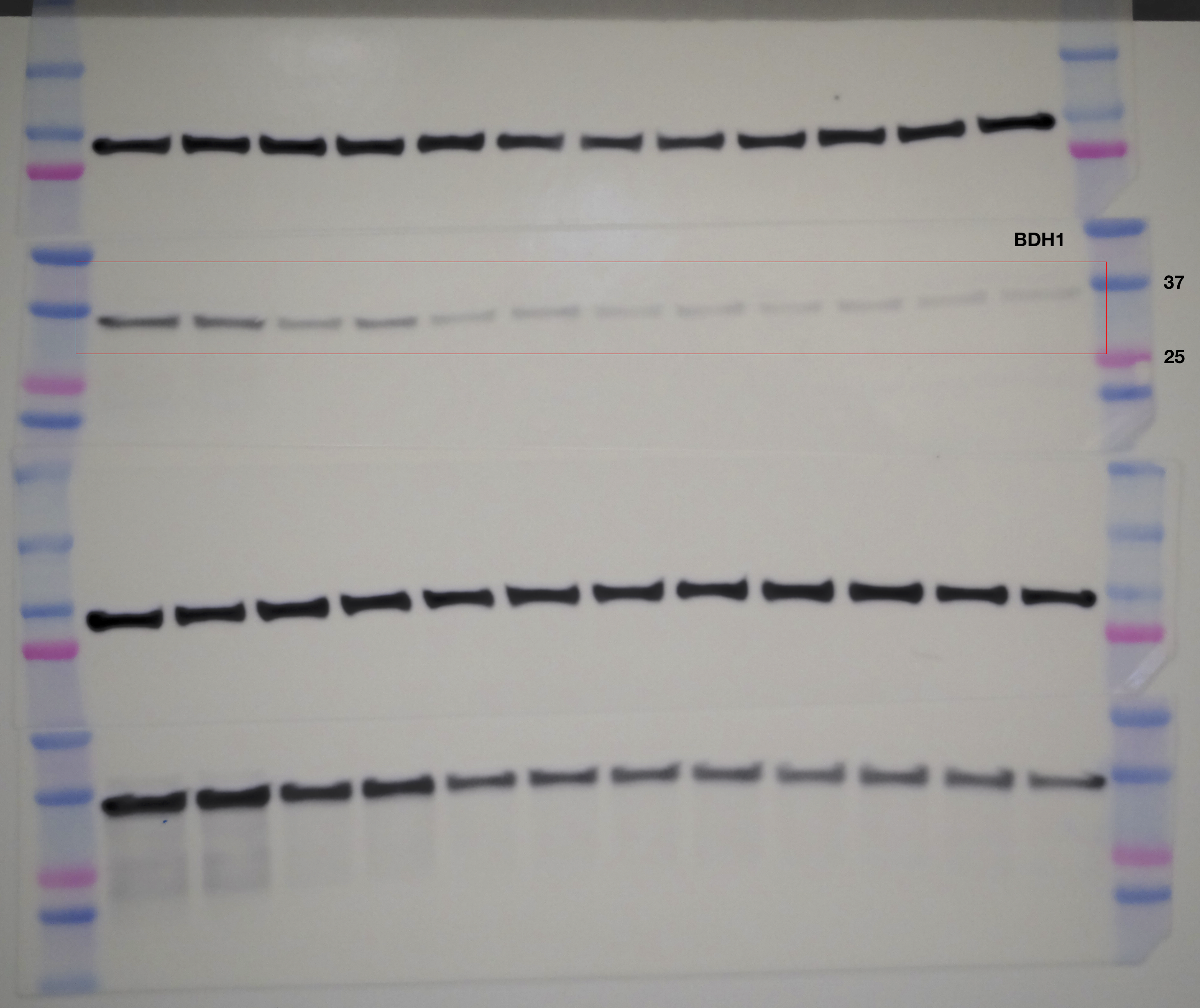

Supplement: Figure 4—figure supplement 1—source data 5. [file elife-106601-fig4-figsupp1-data5.zip › Figure 4-figure supplement 1-source data 5 (S3C)/S3C WT + veh BDH1 .png]

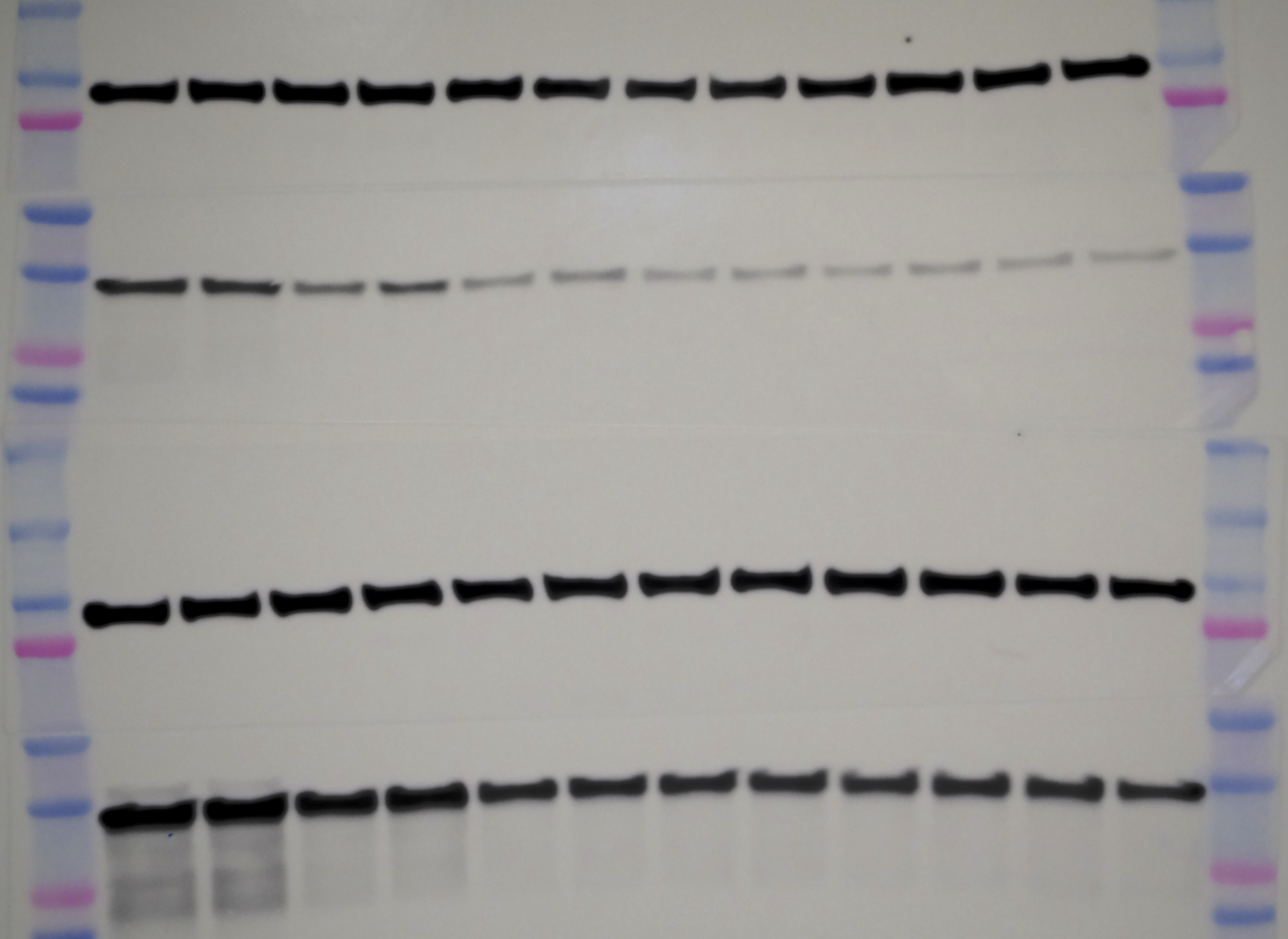

Supplement: Figure 4—figure supplement 1—source data 6. [file elife-106601-fig4-figsupp1-data6.zip › Figure 4-figure supplement 1-source data 6 (S3C)/S3C WT BDH1 veh (p97).png]

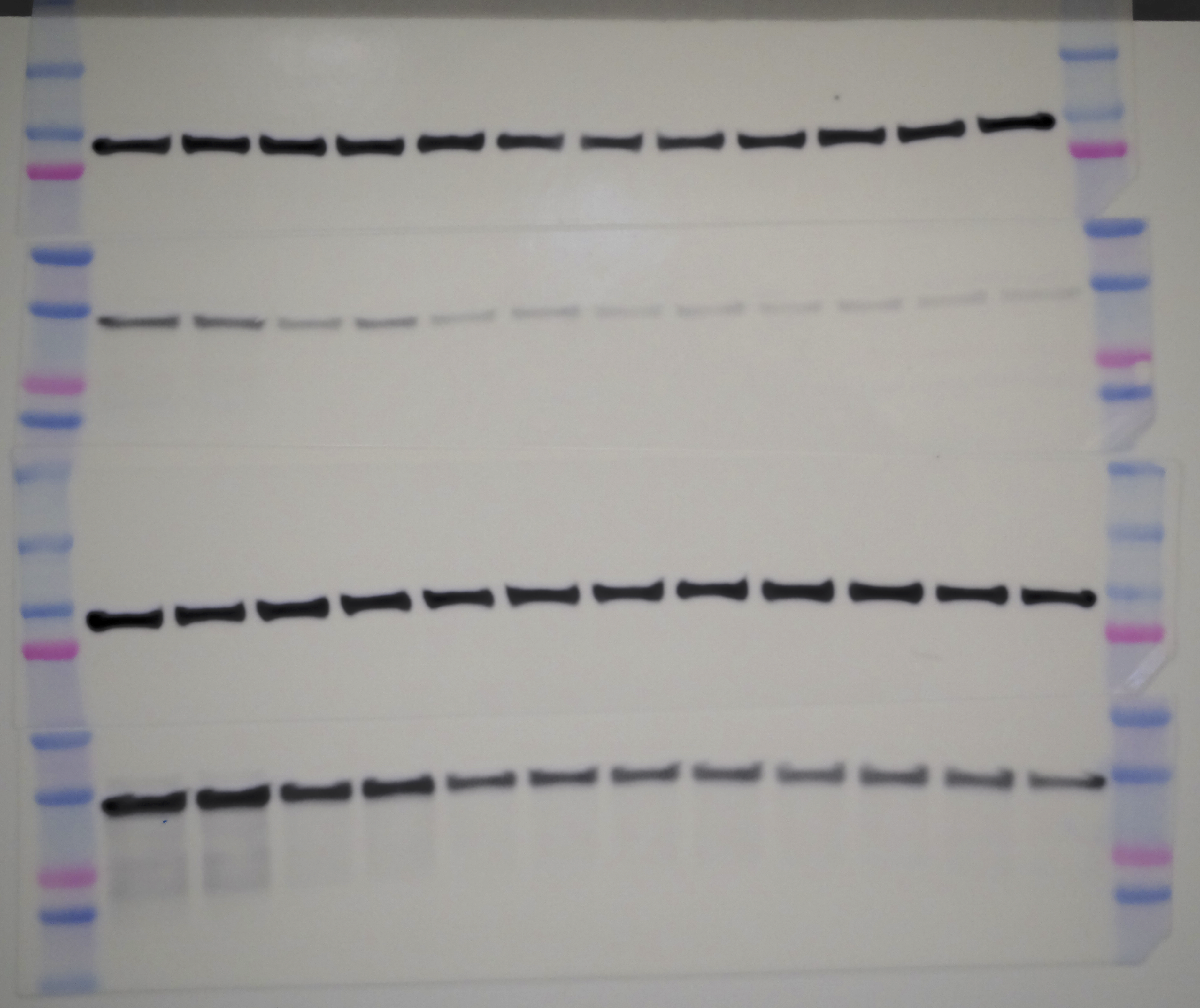

Supplement: Figure 4—figure supplement 1—source data 6. [file elife-106601-fig4-figsupp1-data6.zip › Figure 4-figure supplement 1-source data 6 (S3C)/S3C WT BDH1 veh DSCF1413.png]

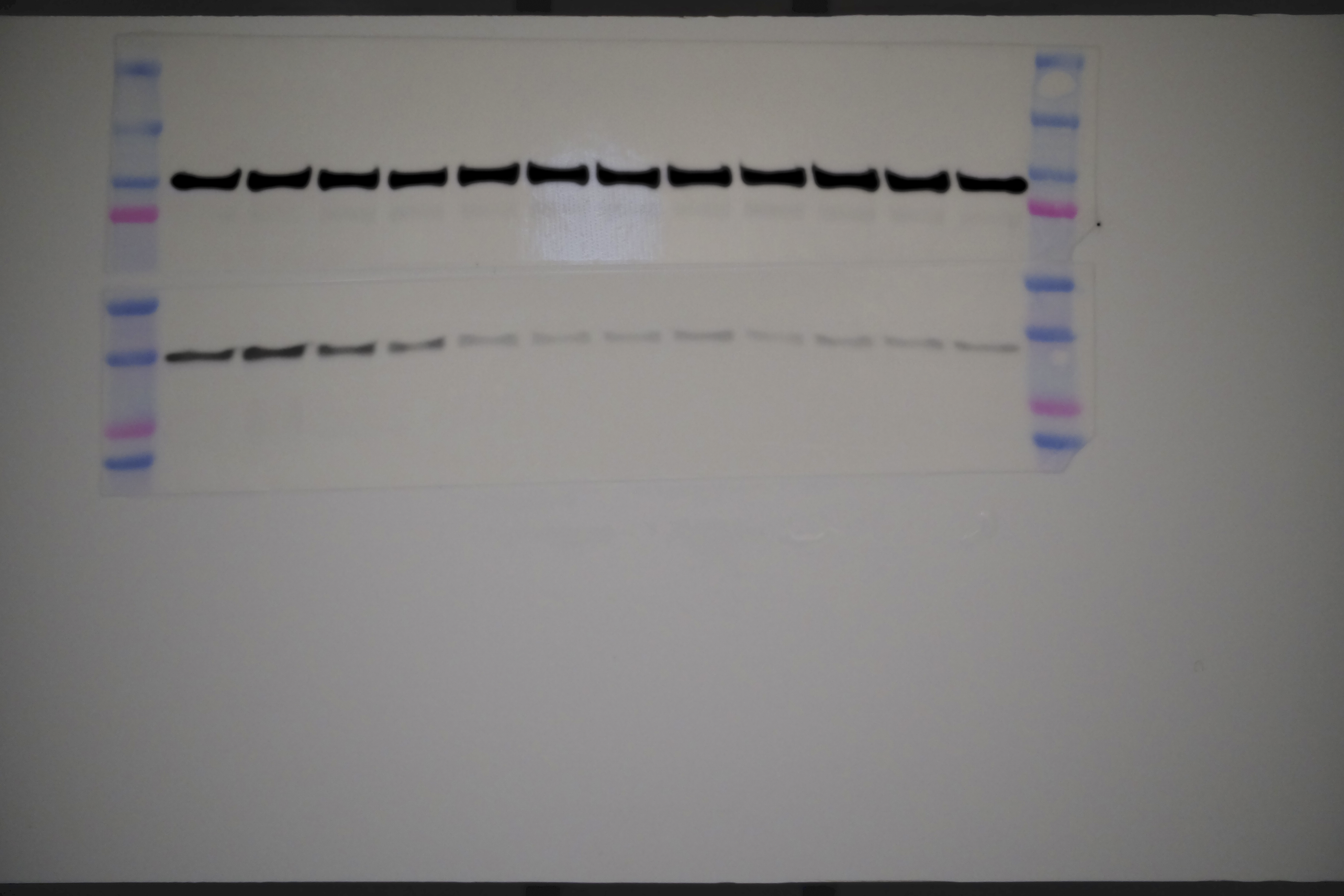

Supplement: Figure 4—figure supplement 1—source data 6. [file elife-106601-fig4-figsupp1-data6.zip › Figure 4-figure supplement 1-source data 6 (S3C)/S3C C115S BDH1 + ECNO.png]

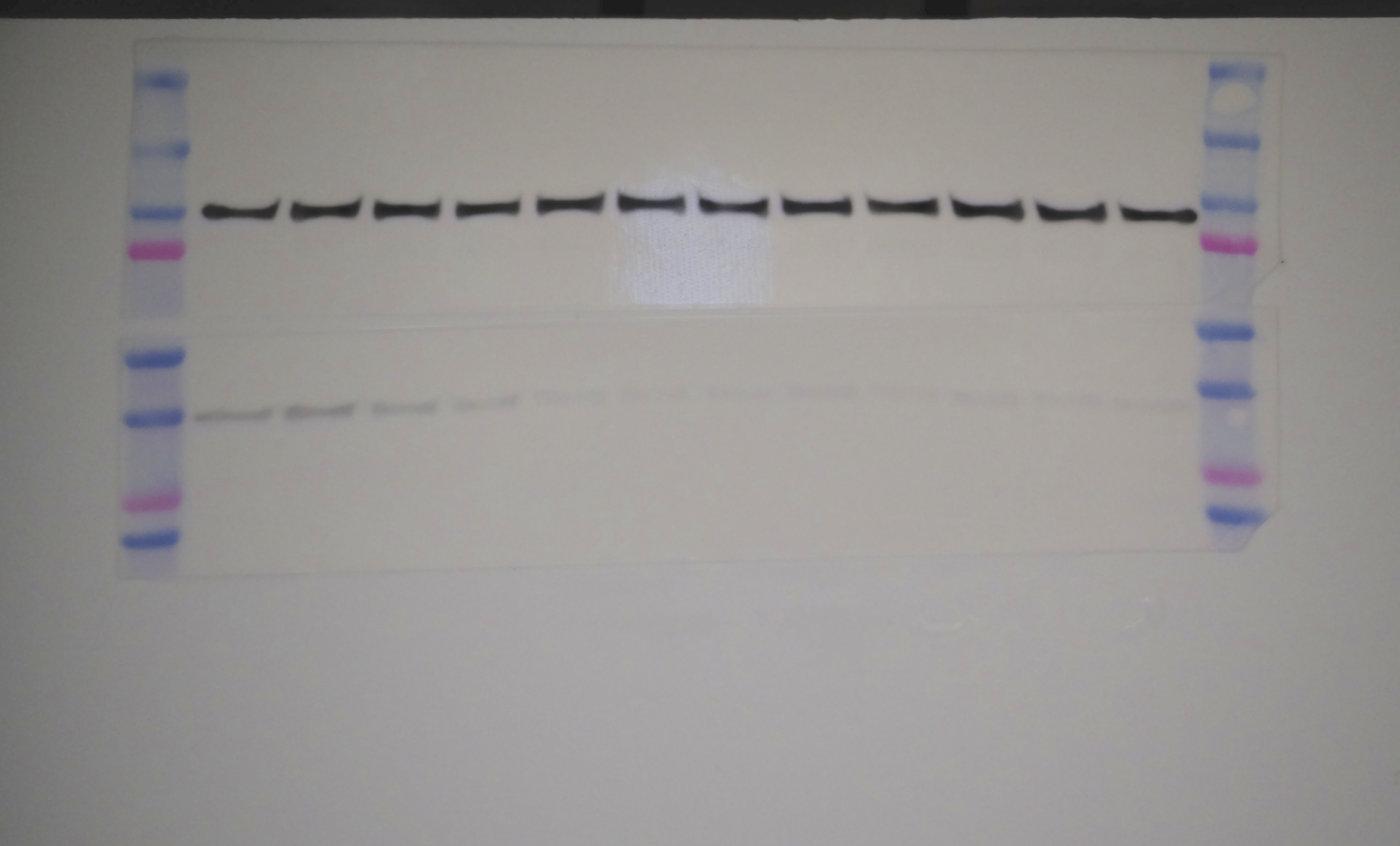

Supplement: Figure 4—figure supplement 1—source data 6. [file elife-106601-fig4-figsupp1-data6.zip › Figure 4-figure supplement 1-source data 6 (S3C)/S3C C115S BDH1 ECNO (just p97, on top).png]

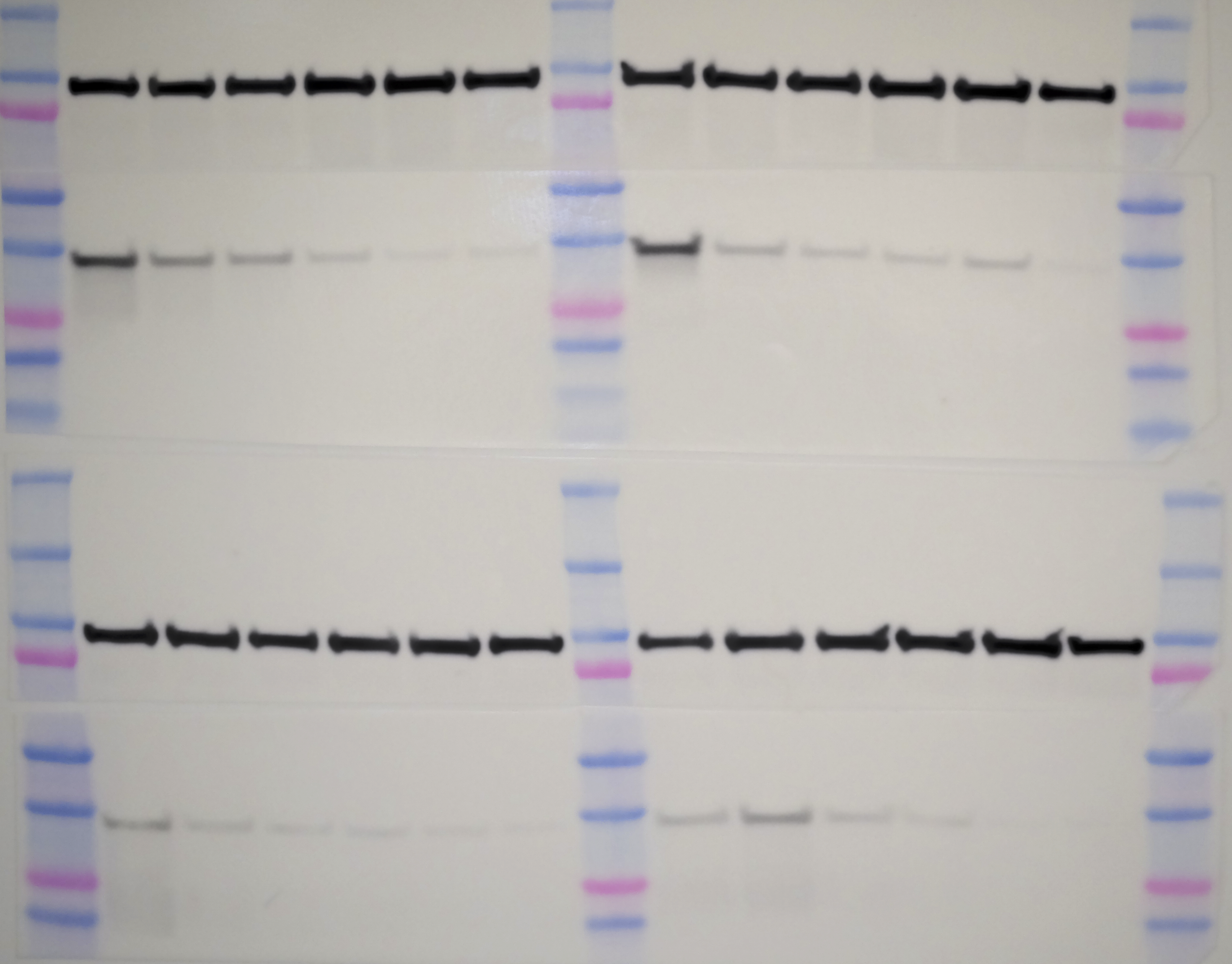

Supplement: Figure 4—figure supplement 1—source data 6. [file elife-106601-fig4-figsupp1-data6.zip › Figure 4-figure supplement 1-source data 6 (S3C)/S3C C115S BDH1 + veh (p97 and BDH1).png]

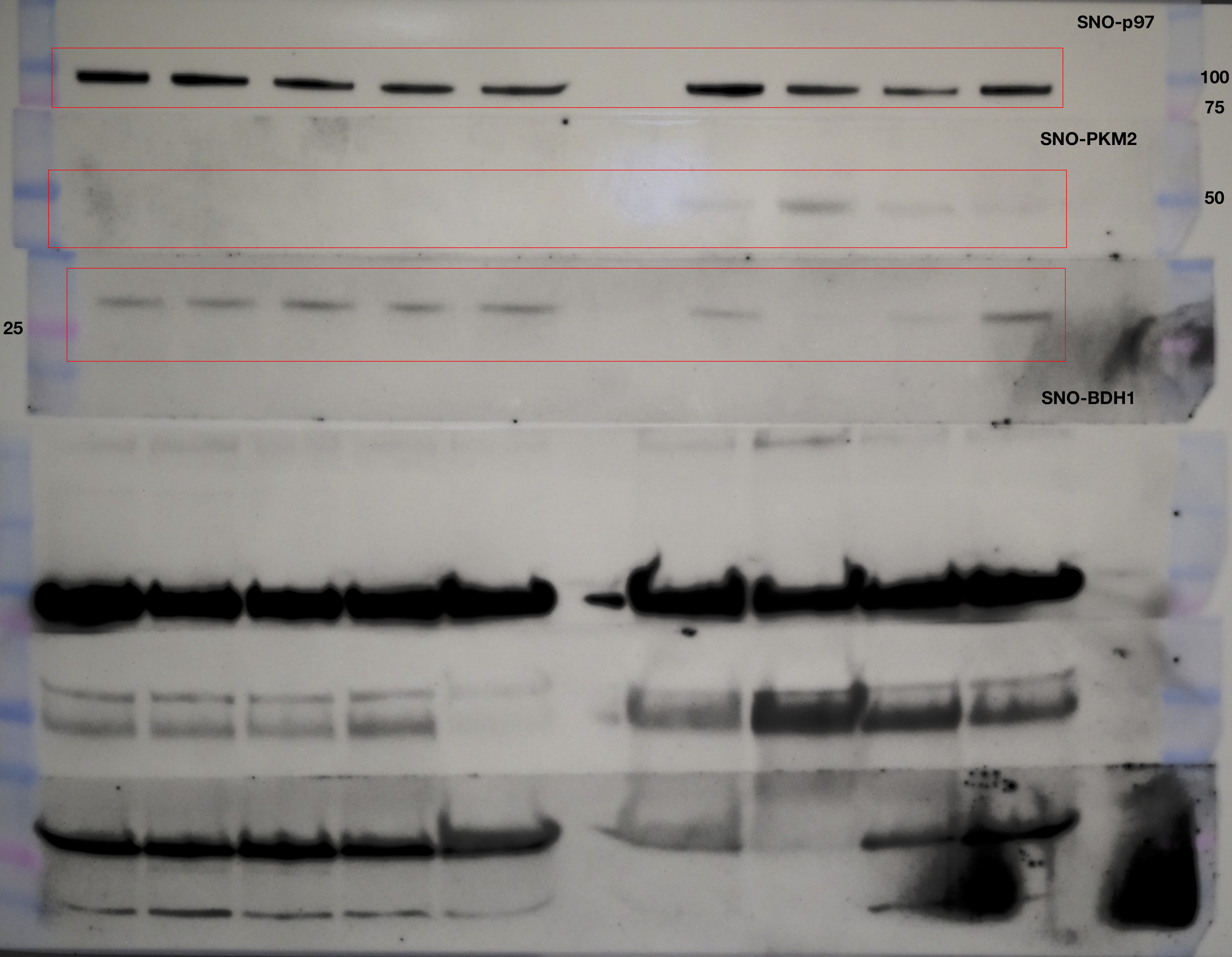

Supplement: Figure 4—figure supplement 1—source data 7. [file elife-106601-fig4-figsupp1-data7.zip › Figure 4-figure supplement 1-source data 7 (S3G)/S3G SNO-BDH1,SNO-PKM2, SNO-p97.png]

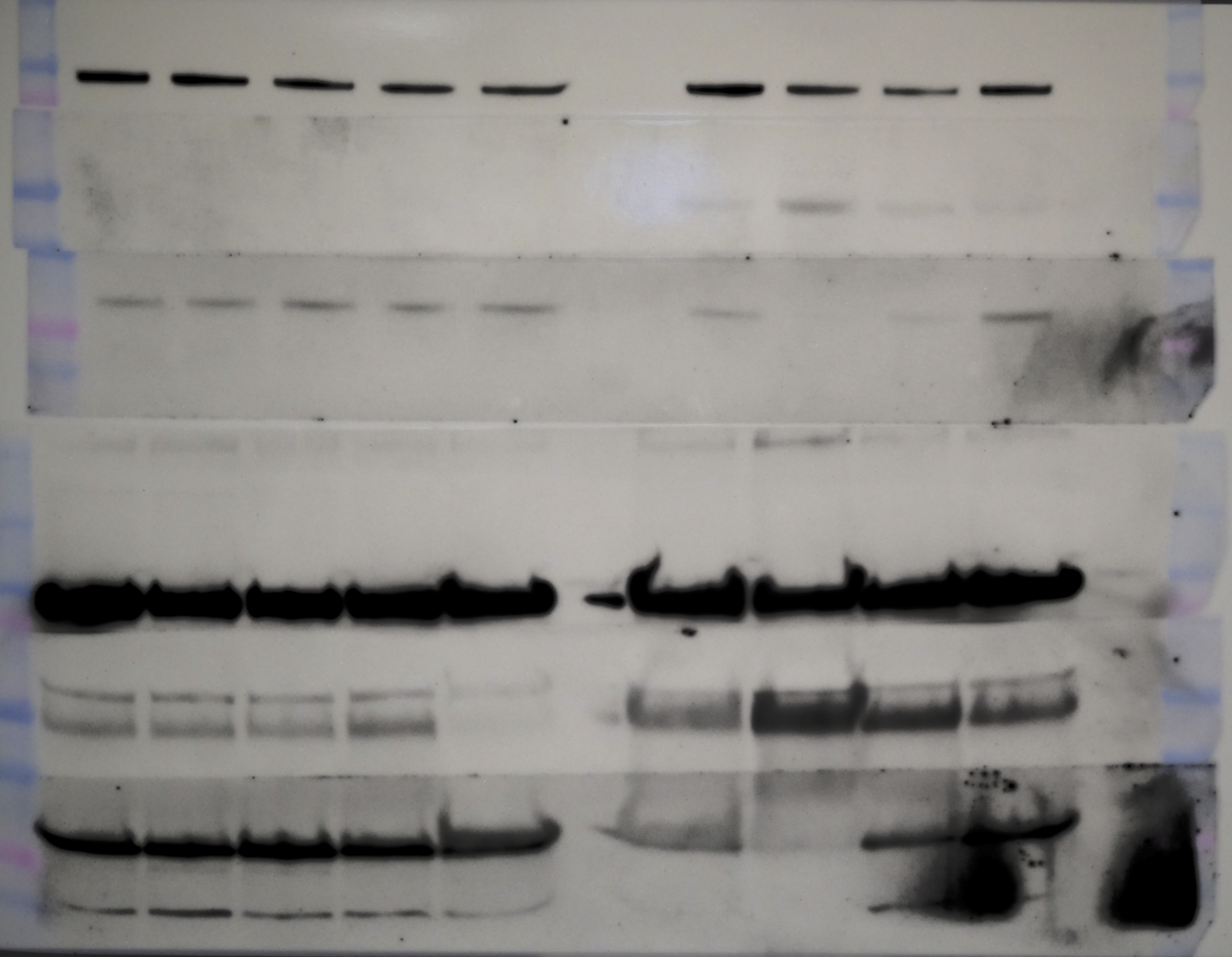

Supplement: Figure 4—figure supplement 1—source data 8. [file elife-106601-fig4-figsupp1-data8.zip › Figure 4-figure supplement 1-source data 8 (S3G)/S3G SNO-BDH1,SNO-PKM2, SNO-p97.png]

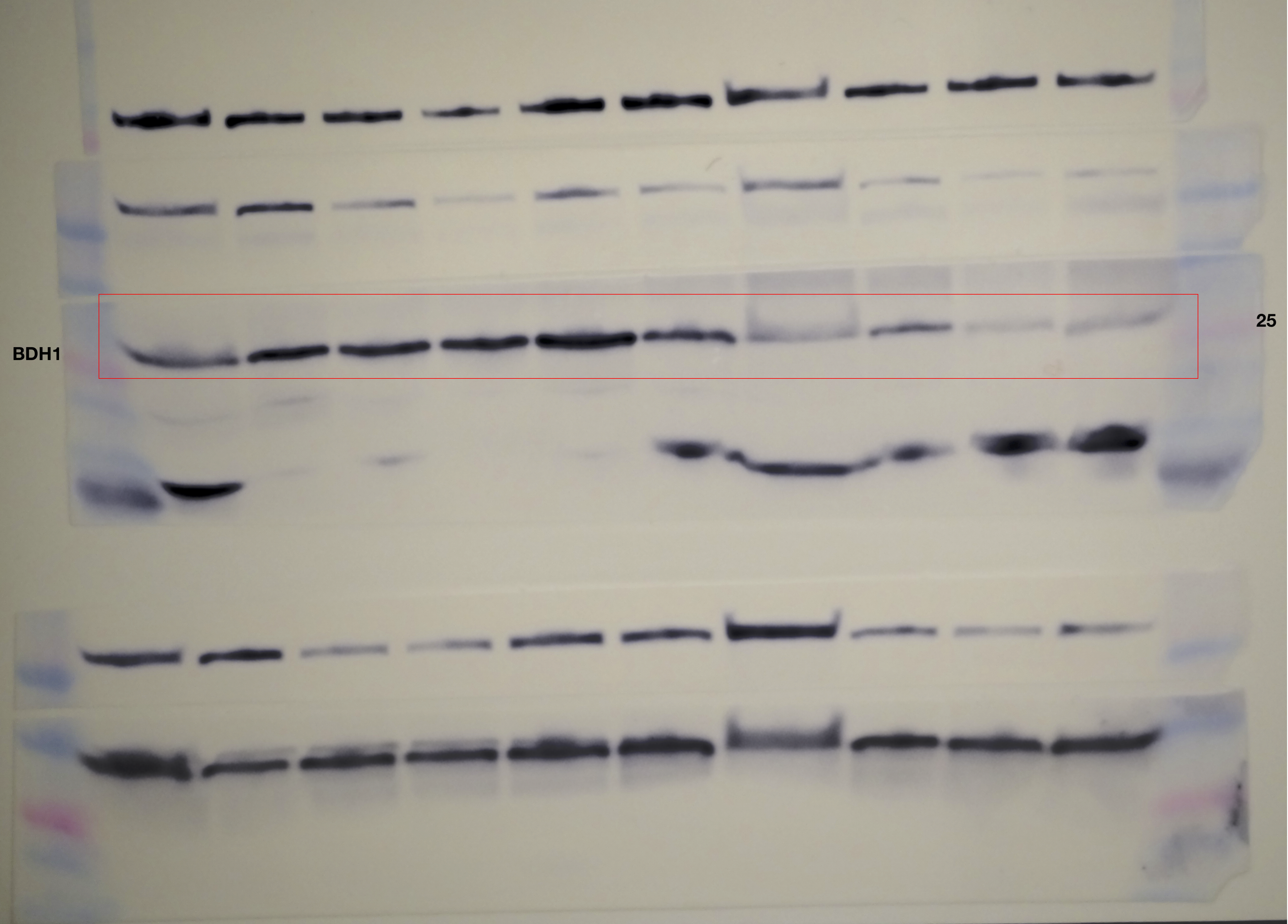

Supplement: Figure 4—figure supplement 1—source data 9. [file elife-106601-fig4-figsupp1-data9.zip › Figure 4-figure supplement 1-source data 9 (S3H)/S3H heart BDH1.png]

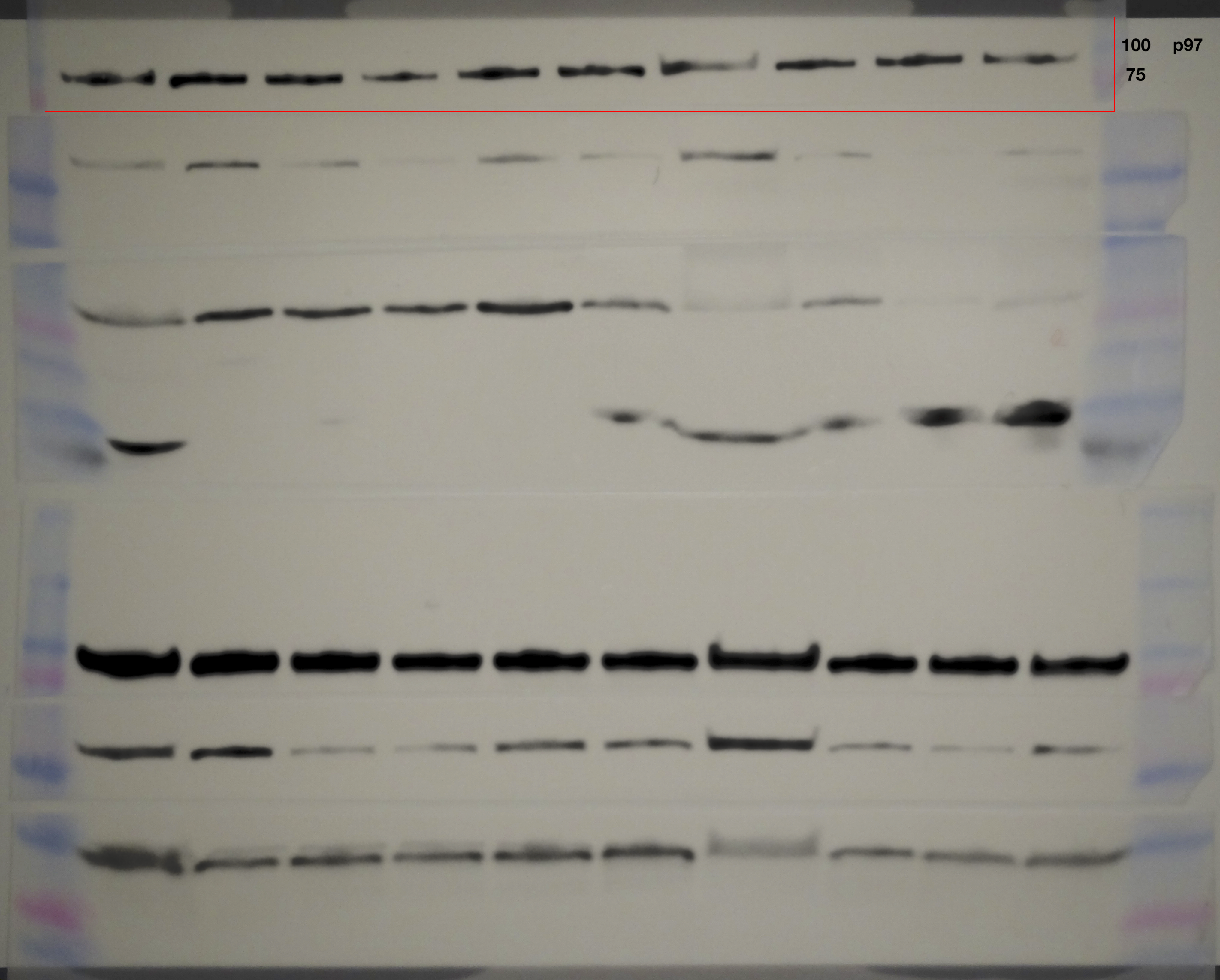

Supplement: Figure 4—figure supplement 1—source data 9. [file elife-106601-fig4-figsupp1-data9.zip › Figure 4-figure supplement 1-source data 9 (S3H)/S3H heart p97.png]

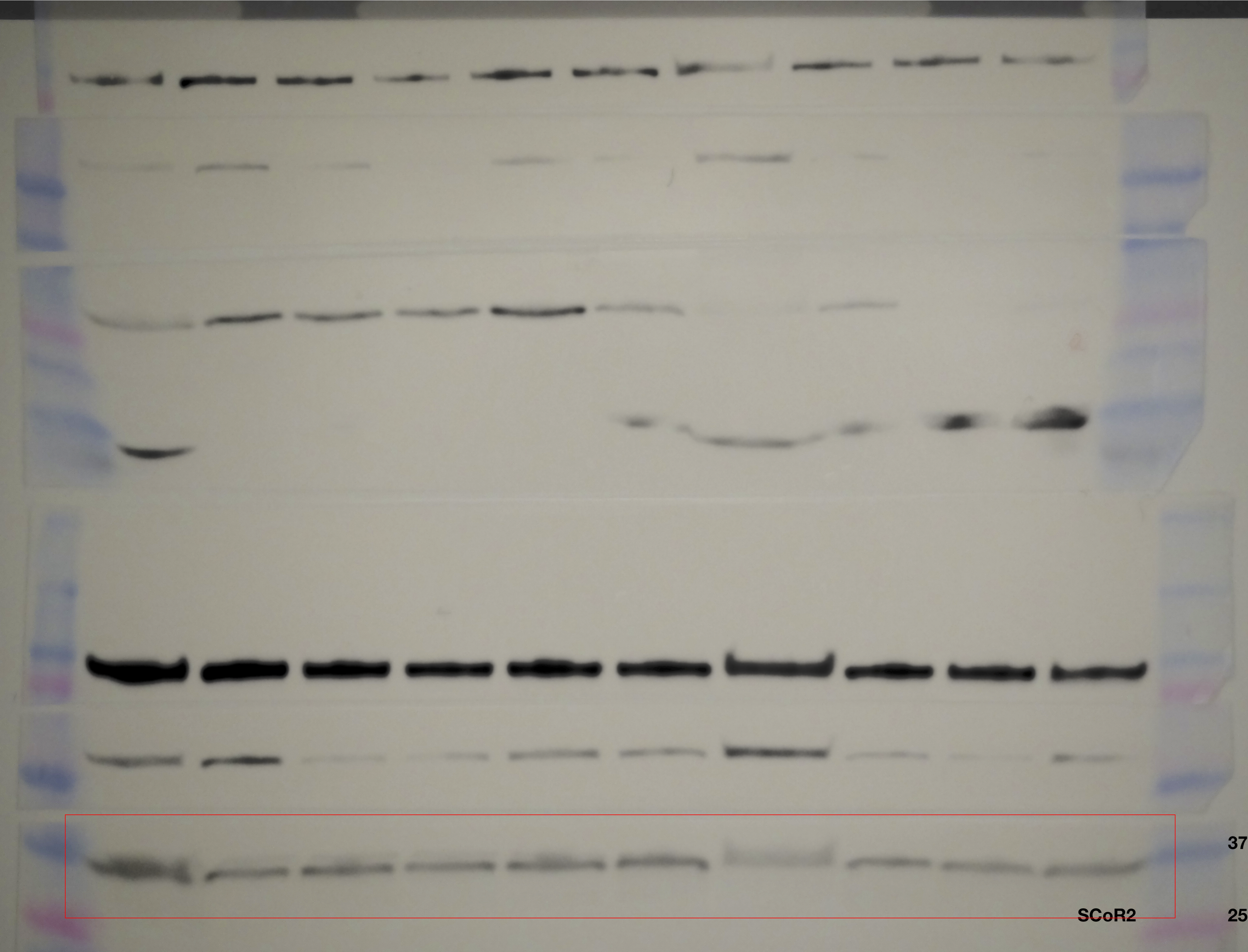

Supplement: Figure 4—figure supplement 1—source data 9. [file elife-106601-fig4-figsupp1-data9.zip › Figure 4-figure supplement 1-source data 9 (S3H)/S3H heart SCoR2.png]

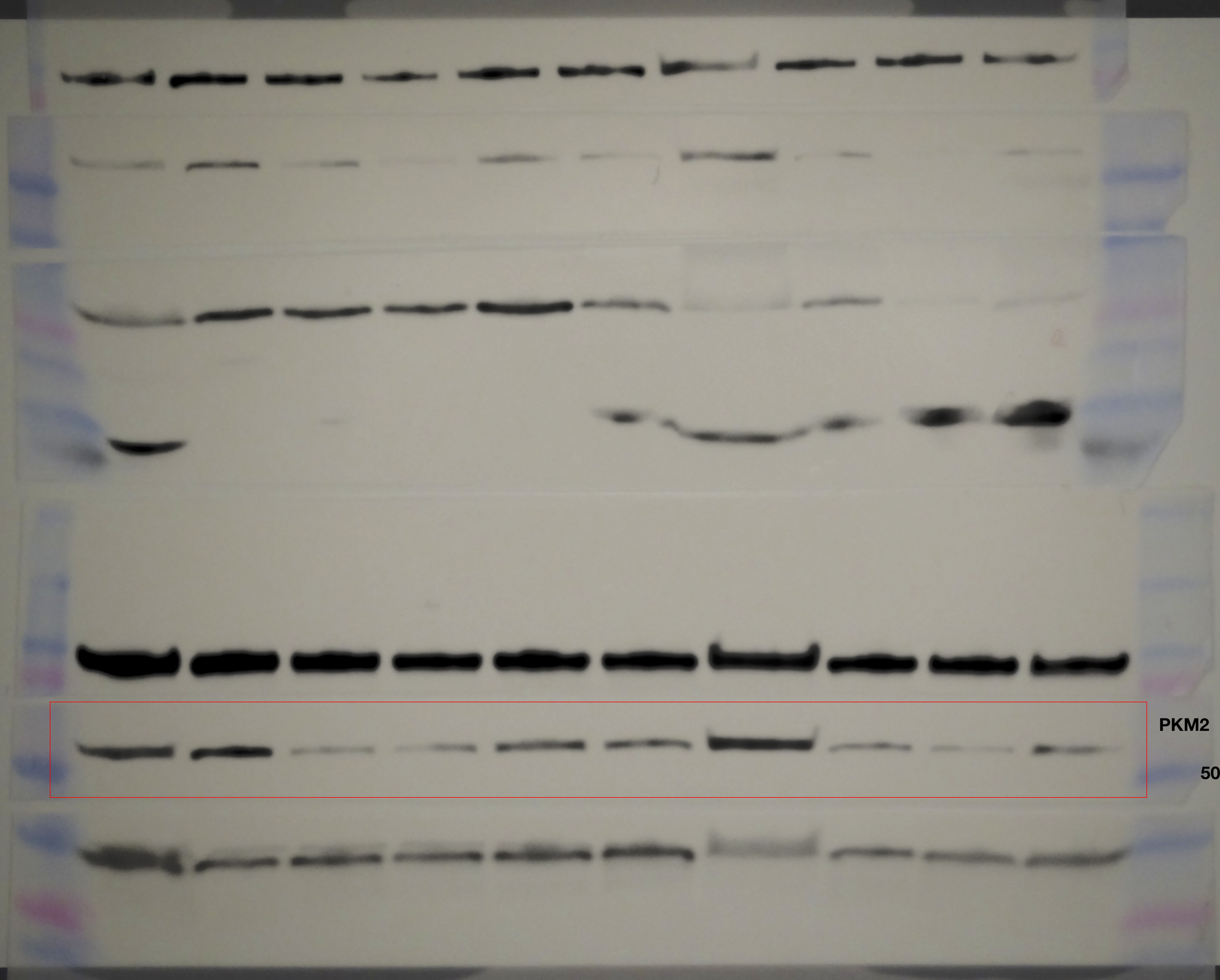

Supplement: Figure 4—figure supplement 1—source data 9. [file elife-106601-fig4-figsupp1-data9.zip › Figure 4-figure supplement 1-source data 9 (S3H)/S3H heart PKM2.png]

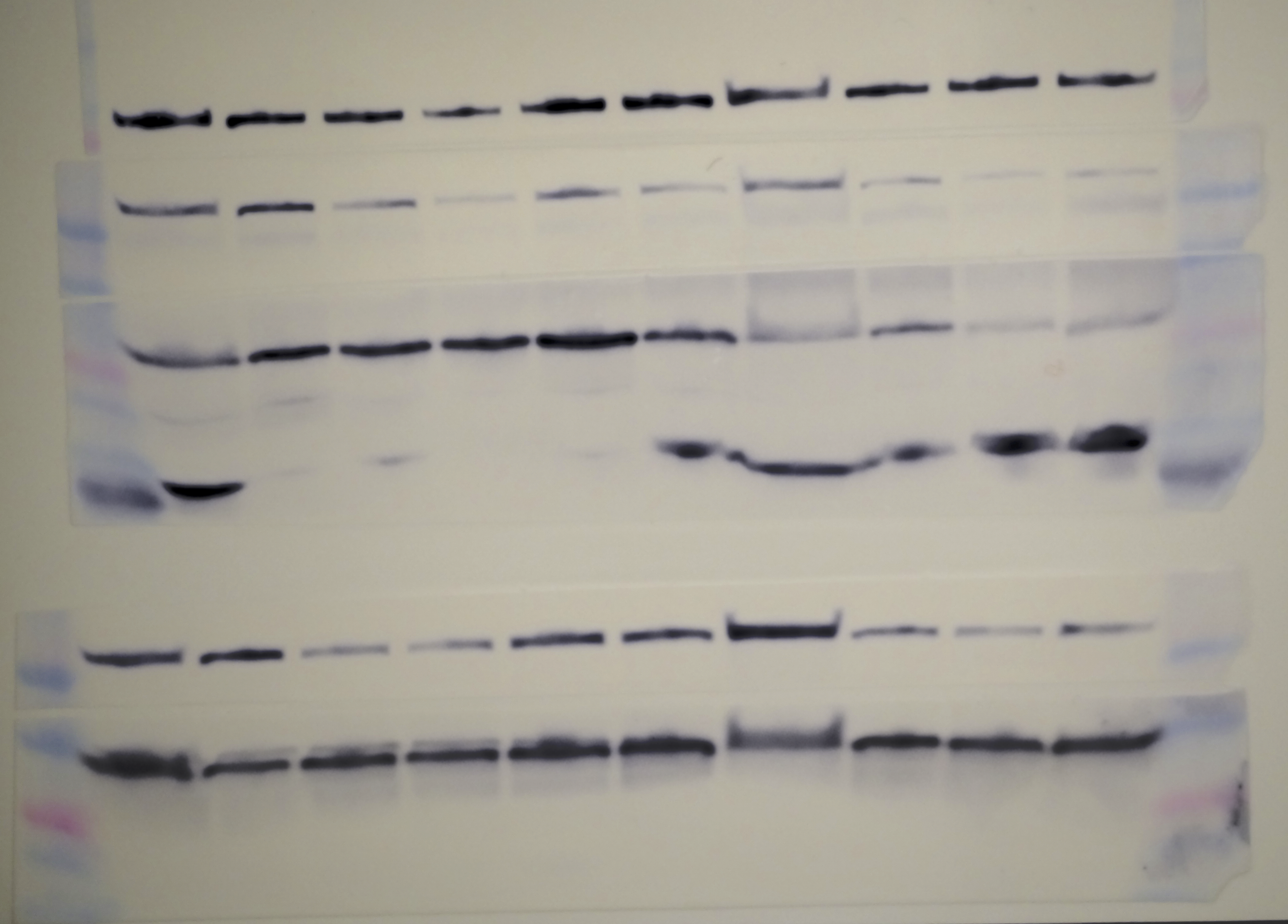

Supplement: Figure 4—figure supplement 1—source data 10. [file elife-106601-fig4-figsupp1-data10.zip › Figure 4-figure supplement 1-source data 10 (S3H)/S3H heart BDH1.png]

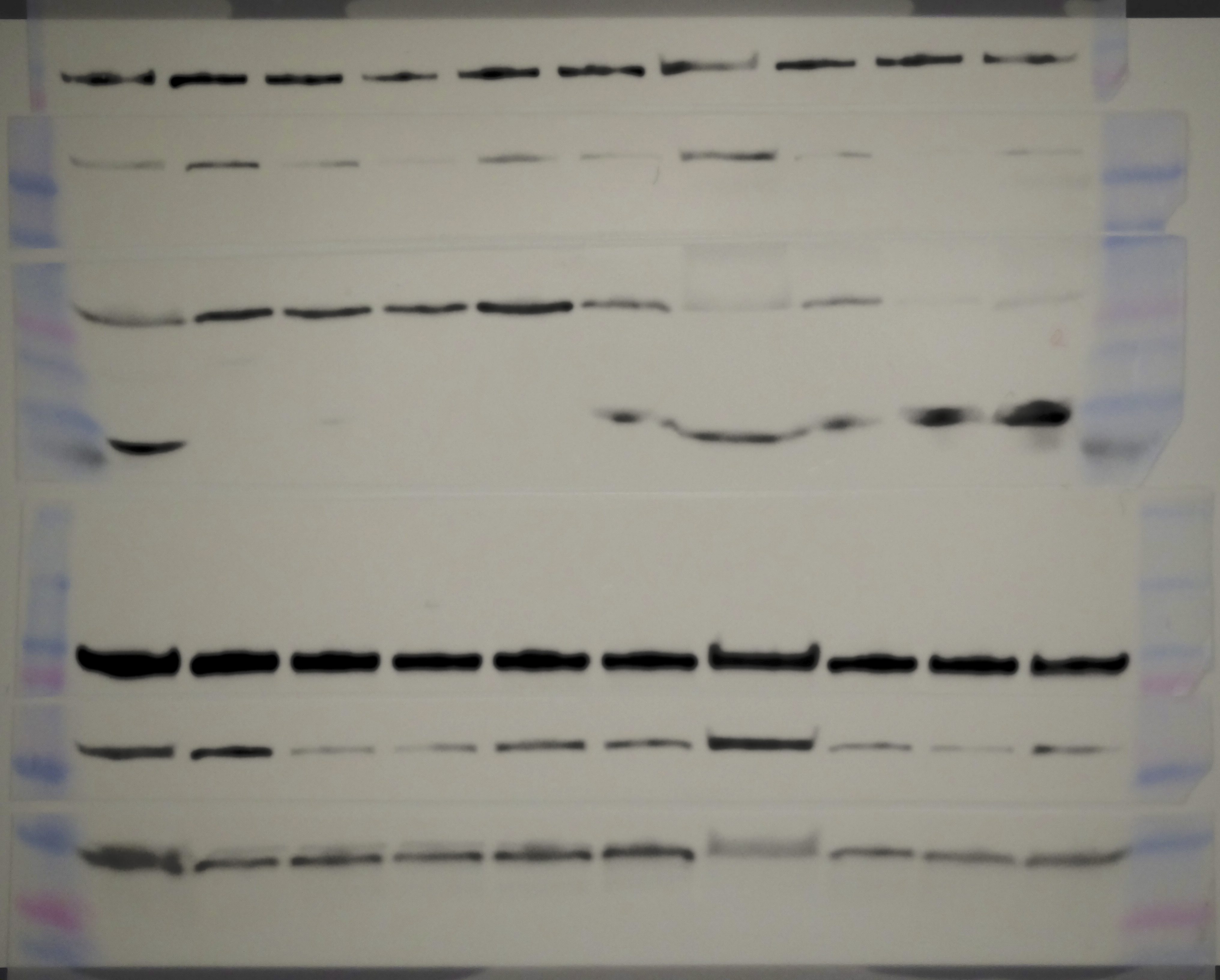

Supplement: Figure 4—figure supplement 1—source data 10. [file elife-106601-fig4-figsupp1-data10.zip › Figure 4-figure supplement 1-source data 10 (S3H)/S3H heart p97.png]

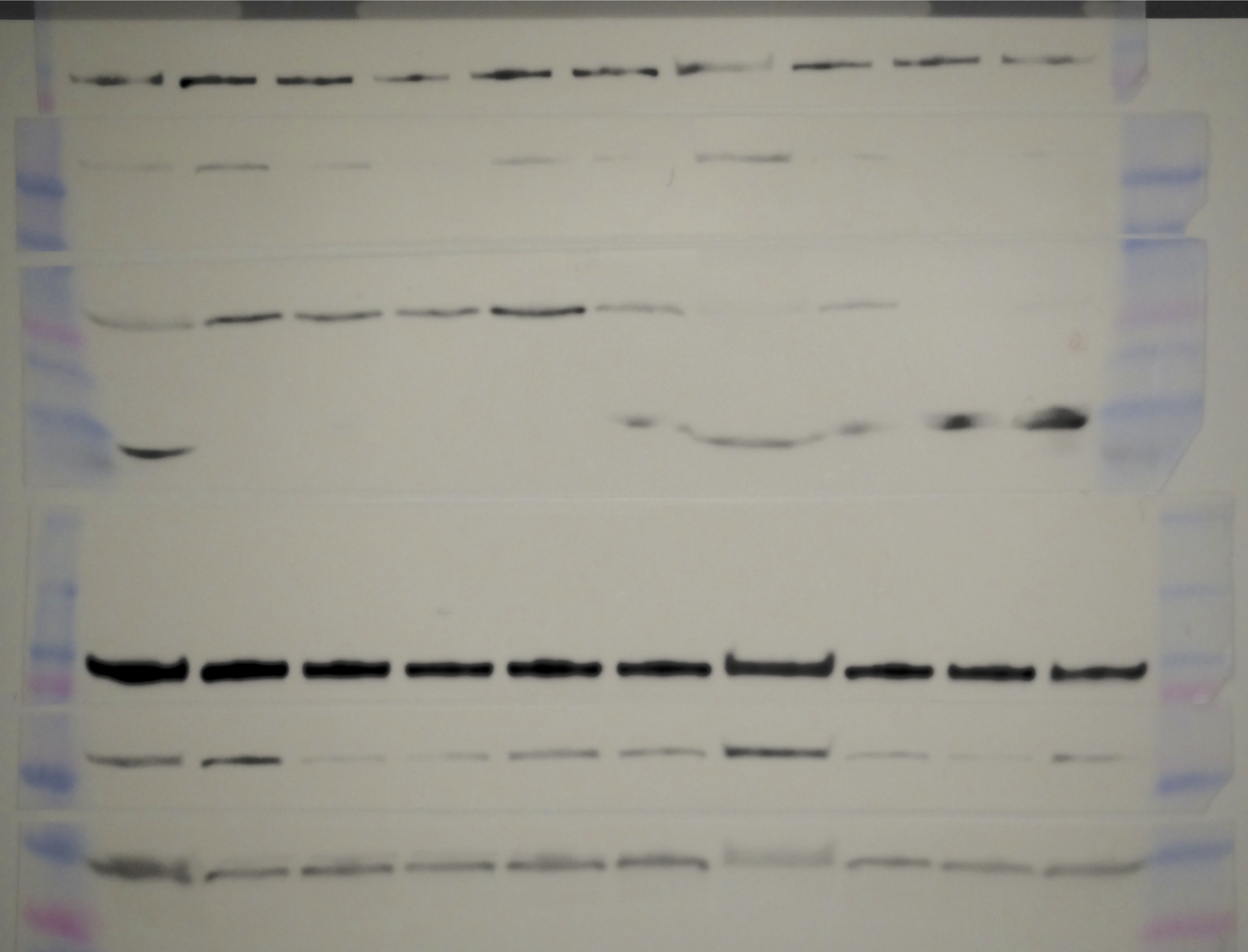

Supplement: Figure 4—figure supplement 1—source data 10. [file elife-106601-fig4-figsupp1-data10.zip › Figure 4-figure supplement 1-source data 10 (S3H)/S3H heart SCoR2.png]
